# N-Acetyl Cysteine May Support Dopamine Neurons in Parkinson's Disease: Preliminary Clinical and Cell Line Data

**DOI:** 10.1371/journal.pone.0157602

**Published:** 2016-06-16

**Authors:** Daniel A. Monti, George Zabrecky, Daniel Kremens, Tsao-Wei Liang, Nancy A. Wintering, Jingli Cai, Xiatao Wei, Anthony J. Bazzan, Li Zhong, Brendan Bowen, Charles M. Intenzo, Lorraine Iacovitti, Andrew B. Newberg

**Affiliations:** 1 Myrna Brind Center of Integrative Medicine, Thomas Jefferson University, Philadelphia, PA, United States of America; 2 Movement Disorders Center, Department of Neurology, Thomas Jefferson University, Philadelphia, PA, United States of America; 3 Department of Neuroscience, Thomas Jefferson University, Philadelphia, PA, United States of America; 4 Division of Nuclear Medicine, Department of Radiology, Thomas Jefferson University, Philadelphia, PA, United States of America; National Health Research Institutes, TAIWAN

## Abstract

**Backgound:**

The purpose of this study was to assess the biological and clinical effects of n-acetyl-cysteine (NAC) in Parkinson’s disease (PD).

**Methods:**

The overarching goal of this pilot study was to generate additional data about potentially protective properties of NAC in PD, using an in vitro and in vivo approach. In preparation for the clinical study we performed a cell tissue culture study with human embryonic stem cell (hESC)-derived midbrain dopamine (mDA) neurons that were treated with rotenone as a model for PD. The primary outcome in the cell tissue cultures was the number of cells that survived the insult with the neurotoxin rotenone. In the clinical study, patients continued their standard of care and were randomized to receive either daily NAC or were a waitlist control. Patients were evaluated before and after 3 months of receiving the NAC with DaTscan to measure dopamine transporter (DAT) binding and the Unified Parkinson’s Disease Rating Scale (UPDRS) to measure clinical symptoms.

**Results:**

The cell line study showed that NAC exposure resulted in significantly more mDA neurons surviving after exposure to rotenone compared to no NAC, consistent with the protective effects of NAC previously observed. The clinical study showed significantly increased DAT binding in the caudate and putamen (mean increase ranging from 4.4% to 7.8%; p<0.05 for all values) in the PD group treated with NAC, and no measurable changes in the control group. UPDRS scores were also significantly improved in the NAC group (mean improvement of 12.9%, p = 0.01).

**Conclusions:**

The results of this preliminary study demonstrate for the first time a potential direct effect of NAC on the dopamine system in PD patients, and this observation may be associated with positive clinical effects. A large-scale clinical trial to test the therapeutic efficacy of NAC in this population and to better elucidate the mechanism of action is warranted.

**Trial Registration:**

ClinicalTrials.gov NCT02445651

## Introduction

Parkinson’s disease (PD) is a devastating neurodegenerative disorder involving the dopamine system that affects more than a million Americans [1]. Standard of care medical treatments for PD are limited to medications that focus on symptom management. Unfortunately, to date no medication has been shown to slow progression in PD. Some supportive therapies such as exercise have shown improved quality of life [2,3], but there is a significant need to continue exploring therapies that might improve symptoms and positively impact the disease process. PD patients often seek adjunct therapies such as dietary supplements, even though most have little to no supportive data [4,5]. Testing those products that have at least a theoretical rationale congruous with what is known of the pathophysiology of PD could have value for patients and providers.

A number of studies have suggested the importance of oxidative stress in the pathophysiology of PD. Oxidative stress itself is defined as a redox imbalance in which there is an excess formation of oxidants or a decrease in the amount of function of natural antioxidants [6]. The brain especially has difficulty withstanding substantial amounts of oxidative stress because of the presence of high amounts of polyunsaturated fatty acids, low levels of antioxidants such as glutathione, and increased iron content in specific areas such as the globus pallidus and the substantia nigra (SN) [7]. In addition, since neurons are in a post-mitotic state, they are unlikely to recover from an oxidative stress insult.

Given the potential importance of oxidative stress in PD, this study focused on n-acetyl cysteine (NAC), which is known to possess substantial antioxidant properties. NAC is the N-acetyl derivative of the naturally occurring amino acid, L-cysteine, and works primarily by helping restore the body’s natural antioxidant, glutathione. NAC is available over-the-counter as an oral supplement and also is available as an injectable pharmaceutical that is primarily used to protect the liver in acetaminophen overdose. We used the combination of oral and IV forms because oral absorption is relatively low (6–10%) and variable [8,9]. Furthermore, an MRS study of 3 patients with PD showed that blood glutathione increased after the start of an NAC infusion and reached a maximum at approximately 60 to 75 minutes [10]. Brain glutathione also increased with maximal values observed at approximately 90 to 110 minutes. Subjects who had the greatest percent change in blood glutathione after NAC infusion also had the greatest percent change in brain glutathione. Interestingly, none of the subjects returned to their baseline brain glutathione levels even at 120 minutes after NAC infusion. Since glutathione itself inefficiently crosses the blood-brain barrier [11], the results of this small study of PD patients suggest that NAC might be useful in increasing brain glutathione levels and thereby impact oxidative processes in the brain.

The goal of the present study was to explore the effects of NAC using both an *in vitro* and *in vivo* approach. To find supportive data for the pilot clinical study, we performed a cell line tissue culture study in which we used a model of PD that employs midbrain dopamine (mDA) neurons generated from human embryonic stem cells (hESCs) [12] to determine whether NAC can protect these mDA neurons from damage resulting from exposure to increasing doses of the PD-like neurotoxin, rotenone. Not only did we hope that this cell line study would be supportive of our clinical trial described below, but it would also corroborate other studies of the protective effect of NAC in animal dopamine cell line studies.

In the clinical study, we measured dopamine transporter (DAT) binding using SPECT with I-123 Ioflupane (DaTscan) before and after receiving NAC for three months. It has been shown that the regional concentration of DAT tends to reflect the tone of the dopamine nervous system in that area [13,14] and is significantly reduced in PD patients [15–17]. DaTscan is approved for clinical use in the US and a number of research studies have shown that DaTscan is able to differentiate PD from controls [18,19]. DaTscan has also been shown to correlate with disease severity [20,21] and has been used in previous trials of PD treatment effects. The Unified Parkinson’s Disease Rating Scale (UPDRS) was also performed on the same day as the DaTscan as a measure of clinical symptoms.

Although this was a pilot study, our goal was to demonstrate whether 1) administration of NAC over three months would result in improved dopamine function as reflected in increased DAT binding on the DaTscan and clinical symptoms as measured by the UPDRS, and 2) confirm that NAC would have a neuroprotective effect in cultures of hESC-derived mDA neurons treated with rotenone.

## Materials and Methods

### Cell Line Study

The human embryonic stem (hES) cell line (H9) (#WA09) were purchased from Wicell (University of Wisconsin, Madison, WI) and maintained according to the supplier’s instructions. Briefly, cells were grown on Geltrex (Life Tech) coated tissue culture plates in mTeSR1 medium (Stem cell Technology). Cell propagation was achieved through manual dissection and transfer of cell colonies every 4 to 5 days. The mDA differentiation process was initiated by passaging them on Geltrex-coated tissue culture plates with two TGF/BMP inhibitors 10 μM SB431542 (Tocris) and 2 μM Dorsomorphin (Tocris), together with 100ng/ml SHH (R&D Systems) and 200nM Purmorphamine (Tocris) for 1 week. Then neural progenitors (NPs) were generated in N2/B27 NEP-basal medium supplemented with FGF8 (R&D Systems) and 20 ng/ml SHH for 1 week. NPs were then expanded in NEP-basal medium supplemented with 20ng/ml bFGF (R&D system) for another week. For further differentiation down the mDA pathway, cells were incubated for 1 week in NEP-basal medium supplemented with 1mM dibutyryl cAMP (Sigma). mDA neurons were pretreated with 10 μM N-Acetyl Cysteine (AllergyResearchGroup) for 3 days and challenged with 15nM, 30nM rotenone (Sigma) for 24 hours. Then cells were fixed and stained for dopaminergic neuron marker tyrosine hydroxylase (TH, 1:200, Pel-freze). Tyrosine hydroxylase is the first and rate limiting enzyme in the production of dopamine. It is highly specific to brain catecholamine neurons. As such, it is the most commonly used marker of midbrain dopamine neurons. Importantly, however, the differentiation protocol used in this study has been previously shown by us [12] and others [22] to give rise to midbrain fated neural progenitors that coexpress both Lmx1a and Foxa2 fate genes and that differentiate into TH+ neurons midbrain dopamine neurons. TH+ cells were counted and averaged over 5 randomly chosen fields/culture in each treatment using Image J in a Nikon confocal microscope. All data was normalized to 100% (untreated control). Unpaired t-test was used for statistical analysis.

### Human Subjects

Written informed consent, approved by the Institutional Review Board of Thomas Jefferson University, was obtained from all subjects and the study was registered on trials.gov with the following identifier: NCT02445651. Of note, there was an administrative delay in registering the IRB approved study (approved on March 13, 2014) on trials.gov until December 2014 The authors confirm that all ongoing and related trials for this drug/intervention are registered. Subjects were recruited from the neurology offices of two co-authors (DK and TWL) who are the co-directors of the Movement Disorder Clinic at Thomas Jefferson University. All subjects were enrolled and had their 3 month follow up between June 26, 2014 and August 13, 2015. Subjects were required to meet the standard clinical diagnosis of PD along with the following inclusion criteria: Age 40–80 years old; physically independent; Hoehn and Yahr score of I-III inclusive; and on stable medication regimen for at least one month. Patients were excluded for the following: Known allergy to iodine or NAC; previous brain surgery; score on Mini-Mental Status examination of 25 or lower; intracranial abnormalities (e.g., stroke, tumor, vascular abnormality); history of head trauma with loss of consciousness > 48 hours; any medical disorder or physical condition that could reasonably be expected to interfere with the assessment of parkinsonian symptoms, or with any of the study assessments including the SPECT imaging; evidence of a significant psychiatric disorder including current alcohol or drug abuse; and female patients who were pregnant or lactating. Subsequently, a full history, physical and neurological examination was performed. Subjects that continued to qualify for the study then underwent an initial DaTscan SPECT study along with qualitative evaluation of their clinical symptoms using the UPDRS.

### NAC Intervention

**Fig 1:**
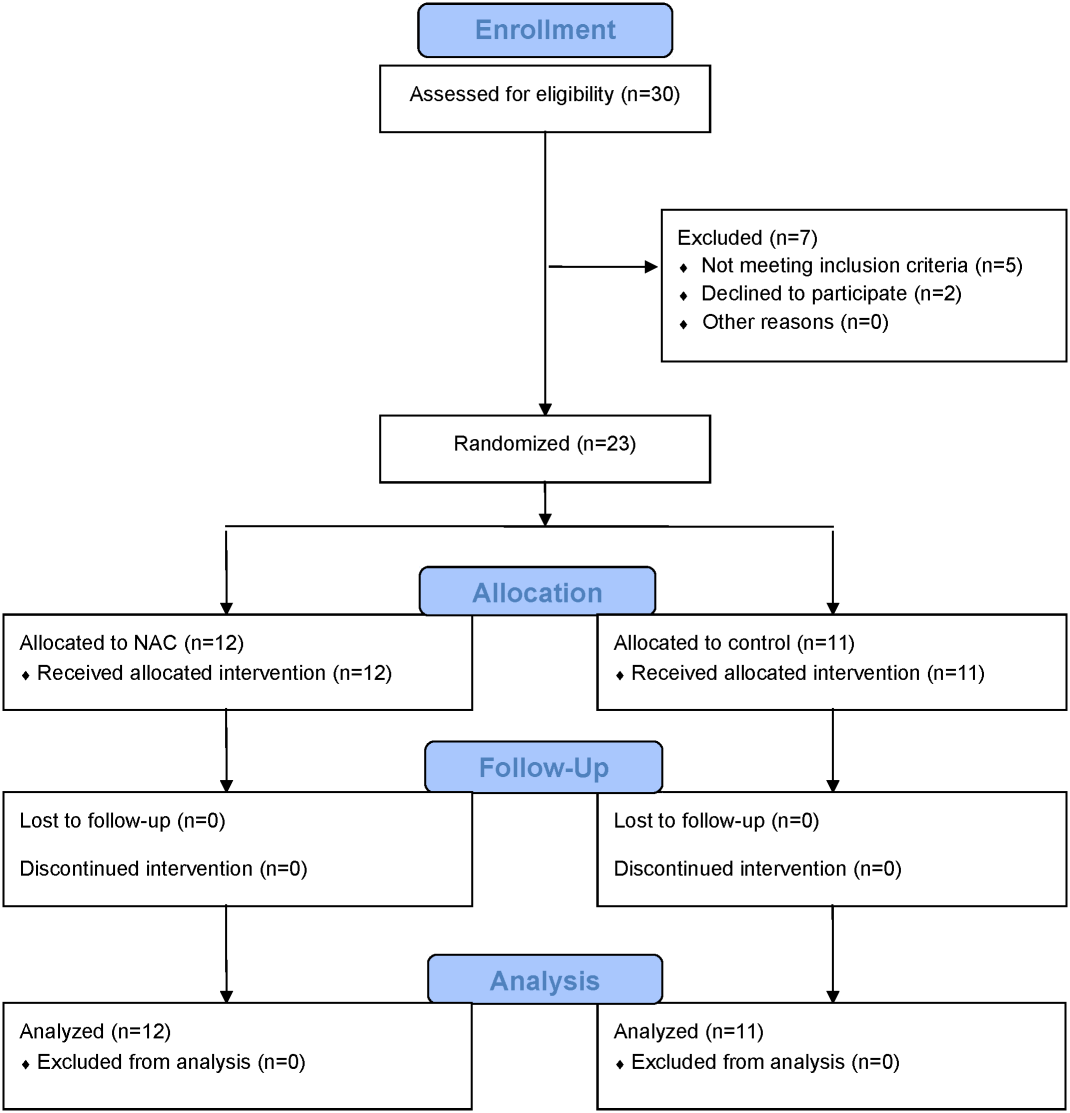
CONSORT Flow Diagram.

Subjects were then randomized using a permuted block method (1:1 ratio using sealed envelopes with the allocation) to either receive intravenous/oral NAC or were placed in the waitlist control condition (see Fig 1). For three months both groups continued their current standard of care PD treatment, with the experimental group receiving NAC, as described below. Additional control subjects included study patients who had an initial SPECT scan 3–6 months prior to entering the study that were compared to the pre-NAC scan.

The NAC was obtained from the Jefferson Pharmacy as Acetadote (Cumberland Pharmaceuticals). Pharmaceutical NAC is an intravenous (IV) medication most commonly used for the treatment of acetaminophen overdose. Acetylcysteine is the nonproprietary name for the N-acetyl derivative of the naturally occurring amino acid, L-cysteine (N-acetyl-L-cysteine, NAC). Acetadote is supplied as a sterile solution in vials containing 200 mg/mL acetylcysteine. The pH of the solution ranges from 6.0 to 7.5. Acetadote contains the following inactive ingredients: 0.5 mg/mL disodium edetate, sodium hydroxide (used for pH adjustment), and Sterile Water for Injection, USP. Acetadote doses were prepared for each patient by a trained study nurse. The dose was 50mg/kg mixed into 200ml of D5W infused over approximately one hour 1x per week. Subjects took the 600mg NAC tablets 2x per day on the days that they did not receive the IV NAC.

After approximately 90 days of receiving oral and IV NAC or being in the waitlist condition, subjects underwent a follow up evaluation, including repeat UPDRS along with the DaTscan SPECT.

### DaTscan SPECT Imaging Procedure

Subjects received a DaTScan before and after completing the NAC for 90 days. Approximately 30 minutes prior to injection of the DaTscan, the patients were given an oral dose of lugol’s solution, which is standard practice for these scans. DaTscan (4–5 mCi, ± 20%) was then injected intravenously through a previously placed catheter. After injection of the DaTscan, the venous catheter was removed. SPECT images were acquired at 3 hours post injection for approximately 45 minutes. This enabled us to obtain qualitative and semiquantitative regional uptake values as determined by our previously described reference region method (see below) [23]. All scans were performed on a Philips Forte gamma camera equipped with ultra-high resolution collimators. All the images were reconstructed using filtered back projection with Chang's first order attenuation correction.

### Data Analysis and Statistics

Manual demarcation of brain regions: A set of standardized templates containing small regions of interest (ROIs) were fit on each DaTscan for the caudate and putamen bilaterally [23]. Within the x-y plane, the ROIs in the template were smaller than the actual structures they represent in order to minimize resolution-induced problems with ill-defined edges. To reduce the effects of volume averaging, the ROIs were not placed on the slices that contained the upper and lower most portions of the structures they represent. The primary outcome measure was the distribution volume ratio (DVR) at 3 to 4 hours post administration, when the distribution of DaTscan has approached a transient, near equilibrium like state that reflects the ratio of k3/k4, which is related to binding potential. This allows for a quantitative assessment of DAT binding. Thus, scanning at this time allows for semiquantitative analysis using a ROI analysis with a background region as an index of nonspecific binding. In addition, an ROI was placed on the midbrain region where DaTscan binding reflects serotonin transporter (SERT) binding as a secondary imaging measure of the effects of the NAC. DaTscan, while not the most specific measure of SERT binding, can reliably evaluate SERT binding due to the non-selective nature of DaTscan and the presence of SERT primarily in the midbrain [24].

Separate linear mixed effects (LME) models with random patient effect were used to model (i) pre- and post-Tx dopamine transporter binding measures from left and right caudate and left and right putamen; (ii) pre- and post-Tx UPDRS measures; and (iii) pre- and post-Tx midbrain serotonin transporter binding measures (Tx refers to NAC plus standard of care of just standard of care without NAC). The LME is adjusted for correlation of the repeated measures from the same patient. The candidate fixed effects predictors included Tx group (NAC vs. Control; N vs. C), age, gender and UPDRS measures in the models for dopamine and serotonin transporter binding measures. The final models were obtained by backward elimination of nonsignificant fixed effects predictors. The association between the pre-to-post-Tx change in UPDRS, dopamine transporter binding in caudate and putamen, and serotonin transporter binding in the midbrain, was evaluated using Pearson correlation coefficients with the corresponding 95% confidence intervals. For confirmation of the DVR analysis, we also performed a paired t-test in Statistical Parametric Mapping software (SPM8; Welcome Department of Cognitive Neurology, University College, London, UK), implemented in Matlab (MathWorks, Natick, Massachusetts). Images were initially normalized using the ioflupane template developed by García-Gómez et al [25]. For our preliminary study, scans from the NAC group were evaluated using a paired t test between the pre and post NAC scans with the threshold set at p<0.05 and minimum of 50 pixel cluster.

## Results

### Cell Line Data

**Fig 2:**
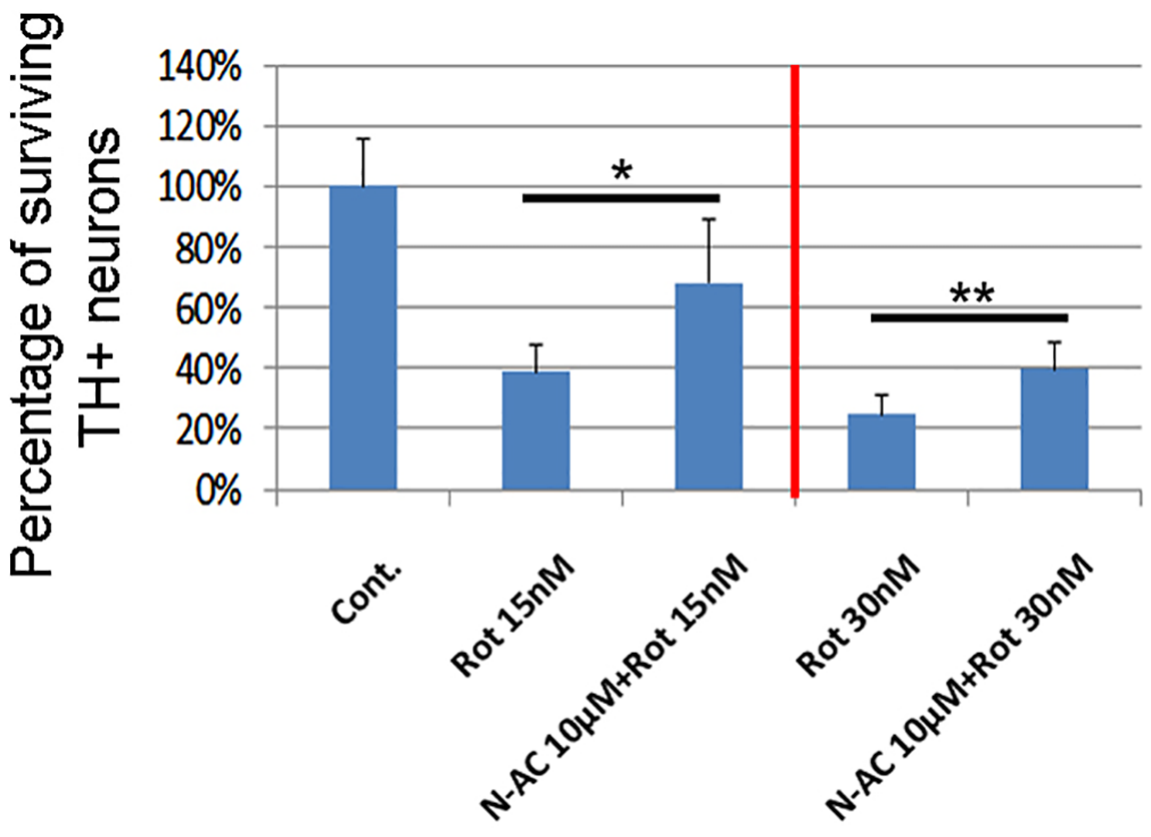
mDA neurons derived from hESCs were treated with N-acetyl cysteine at 10 μM for 3 days before challenge with rotenone (15nM and 30 nM) for 24 hours. TH+ neurons were quantified after cells were fixed and stained. Data was normalized as percentage of surviving mDA neurons compared to untreated control (100%). Treatment groups were compared with rotenone only groups using unpaired t-test. * p<0.05, ** p<0.01.

The results from the cell line study demonstrated that when the mDA neurons derived from hESCs were pretreated with NAC, there was an overall protective effect from both 15 and 30 nM rotenone (see Fig 2). Specifically, the percentage of surviving TH+ neurons after exposure to Rotenone was significantly higher in the NAC treated cells compared to the untreated cells (63% in NAC compared to 39% in untreated for 15nM rotenone and 40% in NAC compared to 22% in untreated for 30nM rotenone).

### Human Study Demographics

For the human subjects study, twenty-three patients with a clinical diagnosis of PD were enrolled for this preliminary assessment, with 12 patients randomized to the NAC arm and 11 patients to the waitlist control arm. There were no patients that dropped out of the study and no significant adverse events. Baseline clinical data for the two groups are presented in Table 1, and were not significantly different.

**Table 1:** Demographics for the study subjects per group.

	NAC Group	Control Group
**Gender**	6M/6F	5M/6F
**Age (Mean±SD)**	59.6±8.2	62.9±7.6
**Duration of PD (mean years±SD)**	3.4±2.2	3.4±1.8
**Hoen and Yahr (Mean±SD)**	1.8±0.5	1.6±0.5
**On/Off Carbidopa/Levodopa**	8/4	6/5

### Imaging and UPDRS Results

**Fig 3:**
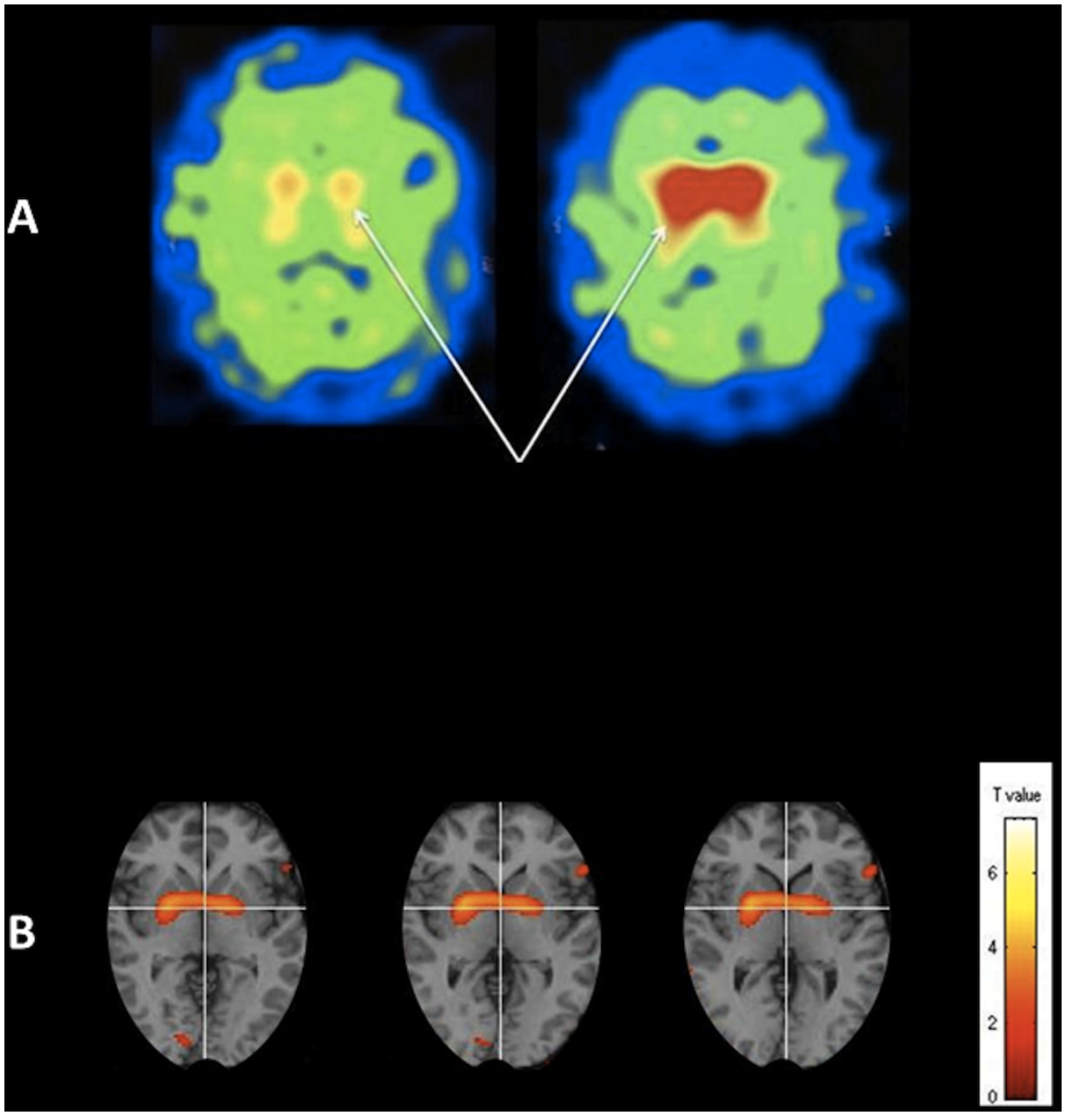
Pre and post NAC DaTscans (left and right respectively) from one particularly responsive patient (A) shows a substantial increase in dopamine transporter binding in the basal ganglia (arrows). We also present (B) the paired t test results overlaid onto a standard MRI template using SPM8 software showing significantly greater binding (p<0.05) post NAC in the basal ganglia.

Although preliminary, there were a number of statistically significant findings that warrant further evaluation in larger clinical trials. Table 2 provides mean pre- and post-Tx dopamine transporter binding measures in each Tx group and each ROI (caudate or putamen). The mean pre-to-post Tx change was positive and significant in the group with Tx = N for both the caudate or putamen (Table 3). In the control group, the mean pre-to-post Tx change was not significantly different from zero for the caudate and significantly negative for the putamen (Table 3). The Pre-to-Post Tx changes in both the caudate or putamen were significantly higher in the Tx group N as compared to controls (Tx group C) (Table 3). The findings are confirmed using the paired t test results in SPM8 (see Fig 3).

**Table 2:** Mean pre- and post-Tx dopamine transporter binding measures.

Group	ROI	Time	Mean	LCL95%	UCL95%
C	caud	pre-Tx	2.85	2.64	3.06
C	put	pre-Tx	1.80	1.59	2.02
C	caud	post-Tx	2.77	2.55	2.98
C	put	post-Tx	1.66	1.45	1.88
N	caud	pre-Tx	2.60	2.40	2.81
N	put	pre-Tx	1.64	1.44	1.84
N	caud	post-Tx	2.75	2.55	2.96
N	put	post-Tx	1.77	1.56	1.97

**Table 3:** Mean pre-to-post-Tx differences in dopamine transporter binding.

Comparison	Mean Difference	LCL95%	UCL95%	p-value
Caud pre-to-Post Tx change in group N	0.15	0.03	0.27	0.014
Put pre-to-Post Tx change in group N	0.12	0.01	0.24	0.039
Caud pre-to-Post Tx change in group C	-0.09	-0.21	0.04	0.171
Put pre-to-Post Tx change in group C	-0.14	-0.26	-0.02	0.027
Caud pre-to-Post Tx change in group N vs. C	0.23	0.06	0.41	0.007
Put pre-to-Post Tx change in group N vs. C	0.26	0.09	0.43	0.003

Table 4 provides estimated pre- and post-Tx mean UPDRS Total Scores in each Tx group. The negative pre-to-post Tx change was significant in the group with Tx = N, but not in the group with Tx = C, and the difference between Tx groups N and C was not significant in terms of the Pre-to-Post Tx changes (Table 5). The model also implies that UPDRS total scores are higher for gender = M as compared to gender = F.

**Table 4:** Mean pre- and post-Tx UPDRS Total Score.

Group	Time	Mean	LCL95%	UCL95%
C	pre-Tx	20.2	15.4	24.9
C	post-Tx	22.2	17.4	26.9
N	pre-Tx	25.6	21.1	30.1
N	post-Tx	22.3	17.8	26.9

**Table 5:** Significant predictors of UPDRS Total Score.

Comparison	Mean Difference	LCL95%	UCL95%	p-value
Gender M vs. F	9.62	3.26	15.99	0.005
Pre-to-Post Tx change in group N	-3.25	-5.33	-1.17	0.004
Pre-to-Post Tx change in group C	-2.00	-4.17	0.17	0.069
Pre-to-Post Tx change in group N vs. C	-1.25	-4.26	1.76	0.397

Although a secondary measure, we also found significant changes in midbrain serotonin transporter binding in the NAC group. The control group essentially did not change going from an initial mean value of 1.53 (95% CI: 1.42,1.63) to 1.51 (95%CI: 1.40, 1.61) while the NAC group went from an initial mean value of 1.48 (95% CI: 1.37,1.58) to 1.65 (95%CI: 1.55, 1.75). The change observed between the NAC group was significant with a p = 0.01 and was also significantly different from controls (p = 0.045). The model also implies that serotonin transporter binding is decreasing with age by 0.008 per year (95%CI: 0.0002, 0.016), consistent with prior studies.

Importantly, there was a significant correlation observed between the change in UPDRS scores and the change in dopamine transporter binding in the caudate (Pearson correlation coefficient of -0.45, CI: -0.73, -0.05, p = 0.026) and putamen (Pearson correlation coefficient of -0.54, CI: -0.78, -0.17, p = 0.006).

## Discussion

A number of studies have suggested the importance of oxidative stress in the pathophysiology of PD. Oxidative stress itself results from either excess formation of oxidants or a decrease in the amount of function of antioxidants [26], which in the case of PD may potentially damage key cellular components such as lipids, proteins, and DNA. Evidence for oxidative damage in the brain of PD patients includes the finding of an increase in the amount of lipid peroxidation products such as malondialdehyde and 4-hydroxynonenal, an increase in protein oxidation as evidenced by protein cross-linking and fragmentation, and an increase in the concentration of 8- hydroxy-2′-deoxyguanosine, a product of DNA oxidation [7].

Additional evidence suggests that reactive oxygen species (ROS) are derived from dopamine itself, which is chemically unstable and undergoes auto-oxidation to form dopamine quinones (DAQs) and superoxide anion radicals. The DAQs can further act as oxidants thus supporting ROS formation. Auto-oxidation of dopamine may be increased in the early stages of PD when dopamine turnover is increased to compensate for dying dopaminergic neurons [27].

Glutathione, an important reducing agent in the neurons, has been found to be depleted in the brain of PD patients [28] and the magnitude of glutathione depletion appears to parallel the severity of the disease and is the earliest known indicator of nigral degeneration, apparently preceding detectable losses in striatal dopamine [29,30]. The brain has difficulty withstanding substantial amounts of oxidative stress because of the presence of high amounts of polyunsaturated fatty acids, low levels of antioxidants such as glutathione, and increased iron content in specific areas such as the globus pallidus and the substantia nigra (SN) [7].

An additional component to the relationship between oxidative stress and PD is related to alpha-synuclein, a prominent component of Lewy body aggregates [31] which are a pathological hallmark of PD. Previous studies have implicated the role of oxidative stress in the formation of synuclein aggregates [32,33]. In addition, several studies have suggested that iron-related oxidative stress can promote α-synuclein aggregation [34,35]. Furthermore, soluble nitrated α-synuclein, which results from interactions with oxidated nitrogen species, appears to activate microglia to produce substantial amounts of ROS through modulation of specific ion channels [36].

Thus, there appears to be growing evidence that oxidative stress likely plays a prominent role in the pathophysiology of PD. When enough oxidative stress occurs, the cell can no longer protect itself resulting in dysfunction and ultimately cell death. The question is whether interventions designed to restore the redox potential will be effective in attenuating the disease process.

NAC is an over-the-counter antioxidant supplement and also is available as an injectable pharmaceutical that protects the liver in cases of acetaminophen overdose. Our cell line study is consistent with other laboratory studies that have suggested how NAC might have a beneficial effect in neurodegenerative disorders such as PD. For example, older studies showed that cotreatment with NAC rescued rat pheochromocytoma cells from the toxic effect of dopamine combined with buthionine sulfoximine, an inhibitor of gamma-glutamyl transpeptidase, or phoron a substrate of glutathione transferase [37]. A more recent study showed that NAC may reduce misfolded protein levels and ameliorate proteotoxicity through heat shock proteins [38]. The authors suggested that their findings broaden the potential mechanisms of action for NAC in neurodegenerative proteinopathies. Another study tested the hypotheses that a combined exposure of nerve cells to oxidative stress caused by hydrogen peroxide and paraquat would elicit synergistic neurodegeneration and that this toxicity would be prevented by NAC [39]. The findings revealed that when neuronal N2a cells received two hits of hydrogen peroxide the result was a severe loss of glutathione that was attenuated by NAC. In fact, NAC reduced the near-complete loss of cells after exposure to dual hydrogen peroxide hits.

NAC can prevent oxidative damage and cell death in an in vitro model that disrupts mitochondrial electron transport function. Thus, NAC could act in vivo against programmed cell death in PD [40]. Long-term treatment with NAC alters NF-kappaB signaling in the brain of mice by increasing cytoplasmic retention of NF-kappaB. This prevents the action of NF-kappaB as a transcription factor in the nucleus [41]. Since increased activation of NF-kappaB may contribute to the pathology in models of Parkinson’s disease, it is possible that the modulating effect of NAC on NF-kappaB activity may be another mechanism by which NAC helps patients with PD [42,43].

Another interesting study showed that NAC is a potent scavenger of both H_2_O_2_ and toxic quinones that are derived from dopamine which can contribute to cell death in PD. NAC also prevented dopamine-mediated inhibition of Na+, K+-ATPase activity suggesting another mechanism for the use of NAC in the treatment of PD [44]. If NAC prevents Na+, K+-ATPase inhibition, it might counteract intracellular damage that leads to dopaminergic neuron death. A study of rat neurons found that NAC reduced methamphetamine induced neurotoxicity in dopaminergic neuronal cells (N27 cells) [45]. Thus, NAC prevents the methamphetamine induced mitochondrial dysfunction and enhanced oxidative stress that induces apoptotic cell death as well as oxidative stress markers.

Administration of NAC has been shown to increase glutathione levels in the mouse brain [46,47]. NAC also has been shown to reduce markers of oxidative damage [48], increase mitochondrial Complex I activity in nerve cells [49], and protect against dopamine cell death from MPTP toxicity [50–52]. In a mouse study, oral NAC was observed to protect dopaminergic terminals against loss related to over-expression of α-synuclein [41]. In α-synuclein over-expressing mice, NAC administration was associated with increased striatal tyrosine hydroxylase positive terminal density and a decrease in α-synuclein immuno-labeling. In addition, NAC administration significantly increased glutathione concentrations in the substantia nigra of mice over-expressing α-synuclein.

Our cell line tissue culture data corroborates the above studies showing that pretreatment with NAC protects mDA neurons derived from hESCs from exposure to rotenone. Thus, this data supports the clinical and DaTscan data in PD patients given NAC. It should also be noted that a major advantage of this approach, compared to other cell and animal studies, is the use of a human rather than rodent cell line model of PD. Moreover, the results can suggest future approaches that could be adapted to test the effects of other molecules on mDA neurons from patient-derived human induced pluripotent stem cells (hiPSCs).

The clinical component of the current study supports other preliminary work. For example, the effect of glutathione in PD was tested in a previous randomized double blind placebo controlled study of 21 PD patients that were not adequately controlled on medication showing a modest effect in symptoms that trended towards significance [53]. We theorized based on the above literature and our own studies that NAC might be more efficient. In addition, an important additional step in the current study was to go beyond a clinical measure such as the UPDRS, and evaluate DAT binding as a physiological marker. Overall, it was highly encouraging to observe substantial changes over such a short period of time, suggesting additional timelines for future studies.

Although a secondary aim, we did find significantly increased SERT binding in the midbrain after NAC supplementation. While some previous studies, including our own, have shown a relationship between SERT binding and depression [54,55], we excluded patients with major depression in the current study, and our patients did not generally report significant depressive symptoms. Thus, the meaning of the SERT binding change is unclear. However, future studies might consider testing NAC in PD patients who specifically express significant depressive symptoms to more carefully screen for depressive symptomatology.

There are several important limitations to the current study. Although we randomized patients and included a control group, it was not a blinded study. It is possible that the improvements that we observed in the NAC group could be related to the placebo effect due to the use of the intravenous injection. Studies have documented a relatively strong placebo effect in Parkinson’s disease patients. For example, in a report by Goetz et al., 11 studies of medical and surgical interventions for PD were evaluated [56]. The authors found that the placebo effect in medication related studies was a mean of 16% with a range from 0–27%. Surgical interventions had a higher mean placebo response of 42%. Future studies comparing NAC to a placebo would help to clarify this issue. In addition, DaTscan and related tracers have been utilized for the evaluation of different therapeutic interventions [57,58] although some concerns have been raised regarding the uniformity of DaTscan findings in multisite trials of different medical interventions in PD [59,60]. However, despite such potential limitations, we felt that DaTscan was an excellent first step for evaluating whether NAC supplementation as described in this study, supports dopamine function in the brain of PD patients. It is also possible that the administration of NAC has a pharmacological effect on the DAT concentration or DAT binding. However, no such effect has been reported and while we observed a mean increased DAT binding in the NAC group, it was not consistent across all subjects.

It should also be noted that the current study provided the NAC as both an IV and oral supplement. It is known that the IV administration of NAC results in substantially higher concentrations of NAC in the plasma. However, it is not known whether these higher concentrations are required for any clinical or physiological effect in PD. Future studies might provide some patients with more IV doses of NAC versus only the oral NAC to determine which route and dose of administration might provide an optimal effect. Overall, this was a pilot study designed to generate hypothesis-testing data that could inform clinical trials adequately powered to assess safety and efficacy [61].

Overall, the current study using both cell line data and human data with DaTscan SPECT suggests that NAC might positively impact dopamine function and potentially clinical symptoms. Future, randomized double blind, placebo controlled trials will be necessary to confirm such an effect in PD patients.

## Supporting Information

S1 Dataset Primary database for image and clinical results. (XLS)

S1 Text CONSORT Checklist. (DOC)

S2 Text IRB approved protocol. (DOC)
