# Supplementary material for: N-Acetyl Cysteine May Support Dopamine Neurons in Parkinson's Disease: Preliminary Clinical and Cell Line Data
Source: PLoS One. 2016 Jun 16;11(6):e0157602. doi: 10.1371/journal.pone.0157602 (PMC4911055; doi:10.1371/journal.pone.0157602)
Supplement: S2 Text — (DOC) [file pone.0157602.s003.doc]

**Physiological Effects of Nutritional Support in Patients with Parkinson’s Disease**

**Principal Investigator: Daniel Monti, M.D.**

**Director, Jefferson Myrna Brind Center of Integrative Medicine**

**Co-Investigators:**

**Andrew Newberg, MD,** Director of Research, Jefferson Myrna Brind Center of Integrative Medicine, Thomas Jefferson University

**Anthony Bazzan, MD,** Jefferson Myrna Brind Center of Integrative Medicine, Thomas Jefferson University

**Daniel Kremens, MD,** Neurology, Thomas Jefferson University

**Tsao-Wei Liang, MD,** Neurology, Thomas Jefferson University

[1.0 INTRODUCTION 4](#__RefHeading___Toc443941782)

[1.1 Introduction 4](#__RefHeading___Toc443941783)

[1.2 Oxidative Stress in PD 4](#__RefHeading___Toc443941784)

[1.3 Use of N-acetyl cysteine in PD Patients 6](#__RefHeading___Toc443941785)

[1.4 Utilizing SPECT to measure the dopamine transporter in PD patients 8](#__RefHeading___Toc443941786)

[1.6 Utilizing Magnetic Resonance Spectroscopy (MRS) to measure changes in the chemical milieu in brain of PD patients 9](#__RefHeading___Toc443941787)

[2.0 OBJECTIVES 10](#__RefHeading___Toc443941788)

[2.1 Evaluate Nutritional Supplementation with intravenous/oral NAC. 10](#__RefHeading___Toc443941789)

[2.1.1 10](#__RefHeading___Toc443941790)

[2.1.2 10](#__RefHeading___Toc443941791)

[2.1.3 10](#__RefHeading___Toc443941792)

[3.0 STUDY PLAN 10](#__RefHeading___Toc443941793)

[3.1 Subject Recruitment 10](#__RefHeading___Toc443941794)

[**3.1.1** **Flow Chart** 12](#__RefHeading___Toc443941795)

[**3.1.2** **Inclusion criteria** 13](#__RefHeading___Toc443941796)

[**3.1.3** **Exclusion criteria** 13](#__RefHeading___Toc443941797)

[3.2 Registration Guidelines and Recruitment 14](#__RefHeading___Toc443941798)

[3.3 Treatment Plan: 14](#__RefHeading___Toc443941799)

[3.3.1 14](#__RefHeading___Toc443941800)

[3.3.2 N-Acetylcysteine 14](#__RefHeading___Toc443941801)

[3.4 Criteria for Removal from / Cessation of Protocol 15](#__RefHeading___Toc443941802)

[3.4.1 Measuring Endpoints: Endpoints 15](#__RefHeading___Toc443941803)

[3.4.2 Subject Withdrawal: 15](#__RefHeading___Toc443941804)

[3.4.3 Missing Appointments: 15](#__RefHeading___Toc443941805)

[3.5 Adverse Events 15](#__RefHeading___Toc443941806)

[3.6 Data Collection and Submission Schedule 16](#__RefHeading___Toc443941807)

[3.6.1 Data Submission: 16](#__RefHeading___Toc443941808)

[3.6.2 Master files 16](#__RefHeading___Toc443941809)

[3.7. Clinical Response 16](#__RefHeading___Toc443941810)

[3.7.1 SPECT Imaging Procedure 16](#__RefHeading___Toc443941811)

[3.7.3 MR Spectroscopy Procedure 17](#__RefHeading___Toc443941812)

[3.8 Statistical Considerations 17](#__RefHeading___Toc443941813)

[3.8.1 Descriptive and Exploratory Analyses: 18](#__RefHeading___Toc443941814)

[3.8.2 Analyses for Primary Objectives: 18](#__RefHeading___Toc443941815)

[3.8.3 Image analysis with SPM: 18](#__RefHeading___Toc443941816)

[Rationale for using SPM. 18](#__RefHeading___Toc443941817)

[SPM analysis. 19](#__RefHeading___Toc443941818)

[Significance assessment. 19](#__RefHeading___Toc443941819)

[3.8.4 Power Analysis: 19](#__RefHeading___Toc443941820)

[3.8.5 Randomization: Randomization will occur via a 1:1 ratio of the,intravenous/oral NAC, or waitlist control groups using the method of random permuted blocks with random block sizes without stratification. 20](#__RefHeading___Toc443941821)

[4.0 RISKS 20](#__RefHeading___Toc443941822)

[4.1 N-acetyl cysteine: 20](#__RefHeading___Toc443941823)

[4.2 Oral NAC/intravenous Supplements: 20](#__RefHeading___Toc443941824)

[**4.2.1** **Supplements, Doses, and Risks** 20](#__RefHeading___Toc443941825)

[4.3 Potential Risks of DaTScan: 21](#__RefHeading___Toc443941826)

[4.4 Special Risk Factors: 21](#__RefHeading___Toc443941827)

[4.5 Risks of venous cannulation: 21](#__RefHeading___Toc443941828)

[4.6 Magnetic Resonance Spectroscopy: 21](#__RefHeading___Toc443941829)

[5.0 REFERENCES 22](#__RefHeading___Toc443941830)

# 1.0 INTRODUCTION

## 1.1 Introduction

The overall goal of this study will be to determine whether NAC will help to support dopaminergic function in patients with Parkinson’s disease (PD). This SPECT study will utilize Ioflupane (DaTscan) single photon emission computed tomography to measure dopamine function, magnetic resonance spectroscopy (MRS) to measure inflammatory and oxidative stress markers, and neurological measures to assess clinical symptoms, in patients with PD who are given oral and IV infusions of NAC which is thought to support the dopamine system’s function.

Parkinson’s disease (PD) is a devastating neurodegenerative disorder of unknown cause that affects more than a million Americans (Korell and Tanner, 2005). Its cardinal features include tremor, rigidity, bradykinesia, and postural instability. Depression is common, and a fourth to a half of all PD patients eventually develop dementia (Shulman et al., 2005). The most prominent PD pathology is degeneration of dopaminergic neurons in the ventrolateral tier of the pars compacta of the substantia nigra in the midbrain, and the formation of α-synuclein cytoplasmic inclusions, termed Lewy bodies, throughout the brain (Fearnley and Lees, 1991). Unfortunately, by the time of clinical diagnosis, substantial neural damage has been wrought, with an 80% reduction in striatal dopamine and a 60-70% reduction of the neurons in the substantia nigra (Bernheimer et al., 2005). It is believed that oxidative stress and inflammation play an important role in the pathophysiology of PD.

The purpose of this study is to evaluate whether NAC that has been shown to have either anti-inflammatory or anti-oxidant effects that might support brain function in patients with PD, particularly with regard to the dopamine system. One arm of this study will receive intravenous and oral NAC, which is a strong antioxidant that increases brain glutathione, that may be beneficial in PD. The second arm will be a waitlist control receiving standard PD therapy. It should be noted that both arms will receive standard PD therapy. One arm will be augmented with the NAC. Since we are not seeking specifically to determine the use of NAC as a treatment for PD, an IND is not required based upon current FDA guidelines.

## 1.2 Oxidative Stress in PD

A number of studies have suggested the importance of oxidative stress in the pathophysiology of PD. Oxidative stress itself is classically defined as a redox imbalance in which there is an excess formation of oxidants or a decrease in amount of function of antioxidants ([[1]](#endnote-2)). The brain has difficulty withstanding substantial amounts of oxidative stress because of the presence of high amounts of polyunsaturated fatty acids, low levels of antioxidants such as glutathione, and increased iron content in specific areas such as the globus pallidus and the substantia nigra (SN) ([[2]](#endnote-3)). Finally, since neurons are in a post-mitotic state, they are unlikely to recover from an oxidative stress insult.

The mechanism by which oxidative stress occurs begins with the production of reactive oxygen species such as superoxide anion (O2−U), hydrogen peroxide (H2O2) and hydroxyl radical (UOH), which are byproducts of metabolism of molecular oxygen by the mitochondria. Excessive formation of reactive oxygen and nitrogen species in PD may damage key cellular components such as lipids, proteins, and DNA. Reactive nitrogen species such as nitric oxide (NO) and its metabolite peroxynitrite (PN) may also play a major role in PD. The “free radical hypothesis” has become prominent in attempting to explain the etiology of PD ([[3]](#endnote-4)). Evidence for oxidative damage in the brain of PD patients includes the finding of an increase in the amount of lipid peroxidation products such as malondialdehyde and 4-hydroxynonenal, an increase in protein oxidation as evidenced by protein cross-linking and fragmentation, and an increase in the concentration of 8- hydroxy-2′-deoxyguanosine, a product of DNA oxidation (Error: Reference source not found).

Additional evidence suggests that reactive oxygen species (ROS) that are derived from the combined presence of dopamine itself, low glutathione, and high iron concentrations are a major cause of dopaminergic cell loss in the brains of PD patients ([[4]](#endnote-5),[[5]](#endnote-6)). Dopamine is chemically unstable and undergoes auto-oxidation to form dopamine quinones (DAQs) and superoxide anion radicals. This reaction is catalyzed by the presence of metals, oxygen, and enzymes such as tyrosinase. The DAQs can further act as oxidants thus supporting ROS formation. Auto-oxidation of dopamine may be increased in the early stages of PD when dopamine turnover is increased to compensate for dying dopaminergic neurons ([[6]](#endnote-7)).

Glutathione is an important reducing agent in the neurons, which is found to be depleted in the brain of PD patients. Glutathione depletion may result from an accelerated loss of glutathione or diminished production. Glutathione is not the only antioxidant molecule reported to be altered in PD, but the magnitude of glutathione depletion appears to parallel the severity of the disease and is the earliest known indicator of nigral degeneration, apparently preceding detectable losses in striatal dopamine ([[7]](#endnote-8),[[8]](#endnote-9)).

Glial cells may also contribute to the problem of oxidative stress in PD even though such cells are protected from toxic levels of H2O2 by possessing high levels of glutathione and glutathione peroxidase that act to detoxify H2O2 to water. However, dopamine is metabolized into H2O2 in glial cells by monoamine oxidases (MAOs). The MAO-B isoform is particularly abundant in the substantia nigra and since the H2O2 produced in glial cells by MAO-B is highly membrane-permeable, H2O2 can cross into neighboring dopaminergic cells. In the dopaminergic cells, H2O2 can react with free iron (Fe++) to produce the hydroxyl radical, which can damage cellular components.

Iron content in the substantia nigra has been found to be elevated in PD compared to age-matched controls. Increased levels of iron and Fe (II) enhance the conversion of H2O2 to the hydroxyl radical via the Fenton reaction. Furthermore, oxidative stress may enhance the levels of free iron via, for example, enhanced release of iron from ferritin by O2. It is not clear whether iron accumulation in the substantia nigra in PD patients is an early or late event and/or whether increased iron helps cause the neurodegenerative process or is a consequence of the pathological process. However, several lines of evidence suggest that iron accumulation along with subsequent oxidative stress may be a primary event in the degenerative process ([[9]](#endnote-10)). For example, a number of iron chelators have been shown to attenuate MPTP toxicity suggesting that iron either mediates or accentuates the associated neuropathological events ([[10]](#endnote-11),[[11]](#endnote-12)). Another study in mice showed that administration of a metal chelator, clioquinol (CQ), in dopaminergic midbrain neurons protected them from MPTP mediated neurodegeneration ([[12]](#endnote-13)). This also resulted in attenuation of motor deficits associated with MPTP in the chelator treated mice.

An additional component to the relationship between oxidative stress and PD is related to alpha-synuclein, a prominent component of Lewy body aggregates ([[13]](#endnote-14)) which are a pathological hallmark of PD. Previous studies have implicated the role of increased oxidative stress in the formation of synuclein aggregates ([[14]](#endnote-15),[[15]](#endnote-16)). Conjugation of dopamine with α-synuclein impedes the usual transition from protoﬁbril to ﬁber resulting in accumulation of more cytotoxic protoﬁbrils in dopaminergic neurons. In addition, several studies have suggested that iron-related oxidative stress can promote α-synuclein aggregation ([[16]](#endnote-17),[[17]](#endnote-18)). Furthermore, soluble nitrated α-synuclein, which results from interactions with oxidated nitrogen species, appears to activate microglia to produce substantial amounts of ROS through modulation of speciﬁc ion channels ([[18]](#endnote-19)).

Overall, there appears to be substantial evidence that oxidative stress, and associated nitrosative stress, likely play a prominent role in the pathophysiology of PD. The normal homeostasis health of a neuron requires adequate maintenance of the redox potential in the cell. When oxidative stress occurs, the cell can no longer protect itself resulting in dysfunction and ultimately cell death. The question is whether interventions designed to restore the redox potential will be effective in attenuating the disease process.

## 1.3 Use of N-acetyl cysteine in PD Patients

NAC is the N-acetyl derivative of the naturally occurring amino acid, L-cysteine. It is a common over-the-counter supplement and also is available as an injectable pharmaceutical that protects the liver in cases of acetaminophen overdose. In the exercise physiology literature, both oral and injectable NAC have been shown to reduce fatigue and improve recovery from exertion ([[19]](#endnote-20),[[20]](#endnote-21)), which has interesting implications for exploring fatigue related to PD.

Laboratory studies have suggested how NAC might have a beneficial effect in neurodegenerative disorders such as PD. For example, one study showed that NAC may reduce misfolded protein levels and ameliorate proteotoxicity through heat shock proteins ([[21]](#endnote-22)). The authors suggested that their findings broaden the potential mechanisms of action for NAC in neurodegenerative proteinopathies. Another study tested the hypotheses that a combined exposure of nerve cells to oxidative stress caused by hydrogen peroxide and paraquat would elicit synergistic neurodegeneration and that this toxicity would be prevented by NAC ([[22]](#endnote-23)). The findings revealed that when neuronal N2a cells received two hits of hydrogen peroxide the result was a severe loss of glutathione which was attenuated by NAC. In fact, NAC reduced the near-complete loss of cells after exposure to dual hydrogen peroxide hits.

NAC can prevent oxidative damage, telomere shortening, and cell death in an in vitro model that disrupts mitochondrial electron transport function. Thus, NAC could act in vivo against programmed cell death in PD ([[23]](#endnote-24)). Long-term treatment with NAC alters NF-kappaB signaling in the brain of mice by increasing cytoplasmic retention of NF-kappaB. This prevents the action of NF-kappaB as a transcription factor in the nucleus ([[24]](#endnote-25)) Since increased activation of NF-kappaB may contribute to the pathology in models of Parkinson’s disease, it is possible that the modulating effect of NAC on NF-kappaB activity may be another mechanism by which NAC helps patients with PD ([[25]](#endnote-26),[[26]](#endnote-27)).

Another interesting study showed that NAC is a potent scavenger of both H2O2 and toxic quinones that are derived from dopamine which can contribute to cell death in PD. NAC also prevented dopamine-mediated inhibition of Na+, K+-ATPase activity suggesting another mechanism for the use of NAC in the treatment of PD ([[27]](#endnote-28)). If NAC prevents Na+, K+-ATPase inhibition, it might counteract intracellular damage that leads to dopaminergic neuron death.

A study of rat neurons found that NAC reduced methamphetamine induced neurotoxicity in dopaminergic neuronal cells (N27 cells) ([[28]](#endnote-29)). Thus, NAC prevents the methamphetamine induced mitochondrial dysfunction and enhanced oxidative stress that induces apoptotic cell death as well as oxidative stress markers.

In animal studies, the administration of NAC has been shown to increase glutathione levels in the mouse brain ([[29]](#endnote-30),[[30]](#endnote-31)). NAC also has been shown to reduce markers of oxidative damage ([[31]](#endnote-32)), increase mitochondrial Complex I activity in nerve cells ([[32]](#endnote-33)), and protect against dopamine cell death from MPTP toxicity ([[33]](#endnote-34),[[34]](#endnote-35),[[35]](#endnote-36)).

In a mouse study, oral NAC was observed to protect dopaminergic terminals against loss related to over-expression of α-synuclein ([[36]](#endnote-37)). In α-synuclein over-expressing mice, NAC administration was associated with increased striatal tyrosine hydroxylase positive terminal density and a decrease in α-synuclein immuno-labeling. In addition, NAC administration significantly increased glutathione concentrations in the substantia nigra of mice over-expressing α-synuclein.

A study of EAAC1(-/-) mice (these animals have neurons with impaired neuronal cysteine uptake, resulting in reduced neuronal glutathione content and chronic oxidative stress), showed substantially reduced neuronal loss when treated with NAC ([[37]](#endnote-38)). The authors suggested that their approach may be a useful model for the chronic neuronal oxidative stress that occurs in PD and that the beneficial effects of NAC might provide an impetus for clinical evaluation of glutathione repletion in PD.

Finally, in humans, an MRS study of 3 patients with PD showed that Blood glutathione increased after the start of an NAC infusion and reached a maximum at approximately 60 to 75 minutes ([[38]](#endnote-39)). Brain glutathione also increased with maximal values observed at approximately 90 to 110 minutes. Subjects who had the greatest percent change in blood glutathione after NAC infusion also had the greatest percent change in brain glutathione. The mean maximal percent change from baseline in brain glutathione was 55% in the PD patients and this was higher than the mean increase in patients with Gaucher’s disease or healthy controls. Interestingly, none of the subjects returned to their baseline brain glutathione levels even at 120 minutes after NAC infusion. The results suggest that NAC might be useful in preventing oxidative damage in PD patients.

This also builds on a related study that investigated the possible direct benefit of intravenous glutathione using a randomized double blind placebo controlled study by Hauser et al ([[39]](#endnote-40)) of 21 PD patients. The patients with PD had motor symptoms that were not adequately controlled on medication. Subjects were randomly assigned to receive intravenous glutathione 1,400 mg or placebo three times a week for 4 weeks. NAC was well tolerated with no difference in adverse events between the placebo and NAC groups. There were no significant differences in changes in Unified Parkinson's Disease Rating Scale (UPDRS) scores. Over the 4 weeks of study medication administration, UPDRS ADL + motor scores improved by a mean of 2.8 units more in the glutathione group (P = 0.32), and over the subsequent 8 weeks worsened by a mean of 3.5 units more in the glutathione group (P = 0.54). This data suggests an improvement that was not maintained consistently after completing intravenous glutathione. The lack of statistical significance was likely due to the small sample size and the confounding factor of different medications taken by patients. The authors point out that the results suggest the possibility of a symptomatic effect, which requires evaluation in a larger study. In addition, a longer treatment period may be required. However, since we are providing the supplements for a 3 month period, subjects would have to receive 36 glutathione infusions (3x per week for 12 weeks). The advantage of NAC is that it can be given both IV and orally so patients only need to have an injection 1x per week while they take the oral NAC every other day. Further, as the evidence above shows, NAC has the ability to increase glutathione levels in the brain.

## 1.4 Utilizing SPECT to measure the dopamine transporter in PD patients

A key component of our program is to evaluate the effects of NAC on dopamine, using SPECT imaging, the relative function of key dopamine-containing regions of the brain in PD patients receiving oral and IV NAC. Specifically, we will plan to study changes in the dopamine transporter (DAT) which reflects the overall health of the dopaminergic system. DAT availability declines with age and declines more rapidly in patients with PD. By measuring the DAT binding before and after receiving approximately 90 days of nutritional support, we can evaluate whether either of the arms receiving supplements helps to support dopamine function.

The DAT mediates the reuptake of free dopamine from the synaptic cleft back into the axonal bouton ([[40]](#endnote-41)). The DAT complex includes a dopamine receptor portion, which is exposed to the intrasynaptic milieu. When free dopamine in the intrasynaptic space binds to the receptor portion, a conformational change occurs in the structure of the transporter complex that physically moves, or “transports”, dopamine across the axonal membrane against a concentration gradient. Once inside the axonal bouton, structurally intact dopamine molecules can then be conserved by being repackaged within the storage vesicles. Alternatively, the level of dopamine, and hence the tone of the dopaminergic system, can be reduced by oxidative enzymes in the cytoplasm that make it unavailable for another transmission. The rate at which dopamine is removed from the synaptic cleft is one of the primary mechanisms for maintaining constant dopaminergic tone. As a result, the regional concentration of transporters tends to reflect the tone of the dopamine nervous system in that area ([[41]](#endnote-42),[[42]](#endnote-43)). This has made the DAT the focus of research in many neuropsychiatric diseases in which dopamine dysfunction has been implicated.

Dopamine transporters may become one of the primary markers for Parkinson’s disease in living patients for several reasons. They are living parts of the neurons that are most affected by the disease, and reflect their health. They are physically coupled with almost all of the diencephalic cells that become dysfunctional. This explains why messenger RNA levels for the dopamine transporter are decreased in the midbrains of patients with Parkinson’s disease. Neuroimaging studies reflect the same phenomenon by visualizing decreased transporter binding in the basal ganglia, that is, the distant regions to which the affected nigral cells project.

Studies of the presynaptic dopamine transporters in patients with symptomatic Parkinson's disease have been consistently positive, regardless of whether PET ([[43]](#endnote-44),[[44]](#endnote-45)) or SPECT ([[45]](#endnote-46)) imaging agents have been used. Exquisite sensitivity may reflect the observation that transporter concentrations decrease more rapidly than the number of axon terminals in animal models ([[46]](#endnote-47)). This tends to keep the intrasynaptic levels of free dopamine constant. If the capacity of the sick neurons to produce dopamine decreases, then reducing the rate at which dopamine is removed from the synaptic cleft tends to move intrasynaptic dopaminergic levels back toward normal. This suggests that the transporter can be an effective marker for studying longitudinal changes in dopaminergic tone, and thus help to assess the effectiveness of interventions to treat PD.

One of the most widely used tracers for the evaluation of the DAT in PD patients is I-123 isoflupane (FP-CIT or DaTSCAN). This tracer is approved for clinical use in the US. A number of research studies have shown that DaTSCAN is able to differentiate PD from controls ([[47]](#endnote-48),[[48]](#endnote-49)) and other related disorders ([[49]](#endnote-50),[[50]](#endnote-51)). DaTSCAN has also been shown to correlate with disease severity ([[51]](#endnote-52),[[52]](#endnote-53)). Finally, DaTSCAN and related tracers have been utilized for the evaluation of different therapeutic interventions ([[53]](#endnote-54),[[54]](#endnote-55)). For these reasons, DaTSCAN is an excellent tracer for evaluating whether oral supplementation as described in this study, supports dopamine function in the brain of PD patients.

Thus, a key component of our program is to evaluate, using SPECT imaging, the relative function of key dopamine-containing regions of the brain in PD patients receiving oral and IV NAC. Specifically, we will plan to study changes in the dopamine transporter (DAT) which reflects the overall health of the dopaminergic system. DAT availability declines with age and declines more rapidly in patients with PD. By measuring the DAT binding before and after receiving approximately 90 days of nutritional support, we can evaluate whether receiving NAC helps to support dopamine function. The SPECT DaTScan is capable of determining the improvements in dopamine function associated with NAC.

## 1.6 Utilizing Magnetic Resonance Spectroscopy (MRS) to measure changes in the chemical milieu in brain of PD patients

It will also be beneficial to assess whether there are specific changes in levels of different molecules that are related to energy consumption and oxidative stress. Proton MR spectroscopy (1H-MRS) has been previously performed in Parkinson's disease (PD) and parkinsonian syndromes to evaluate in vivo concentrations of basal ganglia and cerebral cortex metabolites such as N-acetylaspartate (NAA), choline (Cho), and creatine (Cr) ([[55]](#endnote-56)). This technique has been used to evaluate both the basal ganglia and motor cortex in PD patients ([[56]](#endnote-57),[[57]](#endnote-58)). For example, one study showed a significant reduction in the NAA/Cr ratio in the motor cortex of PD patients compared with controls. Other studies have revealed inconclusive findings in the basal ganglia in PD patients. However, several studies have observed changes in MRS in PD patients in response to therapy. For example, one study demonstrated that after 6 months of therapy with pergolide, PD patients showed an improvement in motor performances and an increase in Cho/Cr ratios in the motor cortex ([[58]](#endnote-59)). The authors concluded that dopaminergic therapy capable of improving motor function might restore the Cho/Cr ratio in the motor cortex. While there are no MRS studies of glutathione concentrations in PD patients, the glutathione level is associated with the level of oxidative stress and has been measured in several other pathological conditions such as head trauma, stroke, and Alzheimer’s disease ([[59]](#endnote-60),[[60]](#endnote-61),[[61]](#endnote-62)). Thus, we plan to include MRS as an additional biomarker to evaluate the potential effects of the nutritional supplements and/or NAC on oxidative stress and cerebral activity in PD patients.

# 2.0 OBJECTIVES

## 2.1 Evaluate Nutritional Supplementation with intravenous/oral NAC.

2.1.1 To evaluate if oral supplementation or intravenous/oral NAC as described below helps to support dopamine function in the brain of patients with PD by measuring clinical symptoms.

2.1.2 To evaluate whether intravenous/oral NAC helps to support dopamine function in the brain of patients with PD by measuring changes in the dopamine transporter using DaTScan SPECT imaging.

2.1.3 To evaluate whether intravenous/oral NAC as described below helps to support changes in oxidative stress and metabolism, as measured by magnetic resonance spectroscopy (MRS), in patients with PD.

# 3.0 STUDY PLAN

## 3.1 Subject Recruitment

Subjects may be pre-screened by telephone using a standardized script and screening form.  Verbal consent and HIPAA Authorization to obtain the prescreening information will be obtained from subjects prior to the prescreening interview. If subjects are prescreened in person, a signed consent and HIPAA Authorization to obtain prescreening information will be obtained. Information collected during pre-screening will be incorporated into the research records as source documentation for subjects included in the study. If subjects are not eligible to participate in the study they will be asked if the information provided in during prescreening maybe retained for consideration in other studies. Prescreening information will be retained for an indefinite period on an official screening form that will be kept in a secure locked area that will only be used by persons involved with research with this research Study.

We plan on recruiting 65 subjects over 3 years with 25 receiving approximately 90 days of combined intravenous/oral NAC, and then 25 subjects who will be in the waitlist control group receiving standard of care to be evaluated initially and then approximately 90 days later. At the conclusion of the study, those in the waitlist control group will be given the option to receive a trial of oral/intravenous NAC if the data suggests that NAC iseffective. All of the study subjects must be clinically diagnosed with PD by a referring neurologist. Subjects may be assessed using structured examinations (i.e., the Unified Parkinson’s Disease Rating Scale (UPDRS) or Movement Disorder Society (MDS) Unified Parkinson’s Disease Rating Scale (MDS-UPDRS) and/or the Hoehn and Yahr scale. Diagnostic criteria will be applied using structured questionnaires, clinical interviews, and neurological examinations. Additional information is gathered, whenever possible, from family members, caregivers, review of previous medical records, and physical and laboratory examination. Subjects may take PD medications, but must be on a stable dose for at least 1 month prior to entering into the study.

To receive a diagnosis of clinically probable Parkinson’s disease, the disease must be progressive, and at least two of the three cardinal signs of PD must be present (bradykinesia, rigidity, and tremor). Additionally, at least two of the following must also exist: (a) asymmetry of signs, (b) asymmetry at onset, (c) absence of characteristics of alternative diagnoses, (d) absence of another etiology known to cause similar features (e.g. neuroleptics).

### **3.1.1 Flow Chart**

**Informed Consent Form**

**Initial Evaluation {N = 65 subjects}:**

Neurological evaluations (MMSE, POMS, PD-39) for PD symptoms

Randomization

**Prescreening**

**Meets all inclusion and exclusion criteria**

NO

**NOT Eligible**

YES

**Eligible**

**Completed Scans:**

SPECT and MRS scans

YES

NO

**Removed from Study**

**Intravenous/Oral NAC Group {N = 25}:**

IV NAC 1x a week plus oral NAC 600mg 2x/day for approximately 3 months until the final evaluation

**Waitlist Control Group**

**{N = 25}:** for approximately 3 months until the final evaluation

**Final Evaluation approximately 90 days into waitlist period:**

DaT scan, MRS scan, and neurological reevaluation to test response

**Final Evaluation approximately 90 days after beginning supplements:**

DaT scan, MRS scan, and neurological reevaluation to test response

### **3.1.2 Inclusion criteria**

1. Clinical diagnosis of PD
2. Age 30-80 years old
3. Physically independent, ambulatory
4. Hoehn and Yahr score of I-III inclusive.
5. On stable antiparkinsonian medication for at least one month
6. Women of childbearing potential will confirm a negative pregnancy test and must practice effective contraception during the period of pilot study. In addition, male subjects who have a partner of childbearing age should practice effective contraception.

### **3.1.3 Exclusion criteria**

1. Known allergy to iodine, cobalt, or NAC that will be given in the study.
2. Previous brain surgery.
3. Score on Mini-Mental Status examination of 25 or lower.
4. Wheelchair-bound or bed-ridden, non-ambulatory.
5. Intracranial abnormalities that may complicate interpretation of the brain scans (e.g., stroke, tumor, vascular abnormality affecting the target area).
6. History of head trauma with loss of consciousness > 48 hours.
7. Any medical disorder or physical condition that could reasonably be expected to interfere with the assessment of parkinsonian syndrome symptoms, or with any of the study assessments including the SPECT imaging.
8. Patients with evidence of a significant psychiatric disorder by history/examination that would prevent completion of the study will not be allowed to participate.
9. Patients with current alcohol or drug abuse
10. Pregnant or lactating women.
11. Enrollment in active clinical trial/ experimental therapy within the prior 30 days.
12. Pending surgery during the course of the study.
13. [History of very low blood pressure](http://www.webmd.com/heart/understanding-low-blood-pressure-basics).
14. [History of thrombocytopenia](http://www.webmd.com/a-to-z-guides/thrombocytopenia-symptoms-causes-treatments) or clotting disorders.
15. Cancer patients receiving active chemotherapy.
16. History of active gallstone problems or a bile duct obstruction.
17. Severe gastroesophageal reflux disease.
18. History of uncontrolled diabetes, asthma, gastroesophageal reflux disease, thyroid conditions.
19. History of severe kidney disease (if a patient reports this problem, a serum creatinine will be checked to assess GFR and if it is less than 30, they will be excluded),
20. History of Leber’s disease, a hereditary eye disease.
21. History of uncontrolled hypercalcemia
22. History of active sarcoidosis, histoplasmosis, or lymphoma.
23. Patients taking medications that might interact with NAC involved in this study will be evaluated on a case by case basis by the PI or study physician. These medications include: Medications for high blood pressure; Medications that slow blood clotting; Medications for diabetes; Nitroglycerin.

## 3.2 Registration Guidelines and Recruitment

Study subjects initially will be recruited by referral from the Jefferson Movement Disorders Clinic and local neurology groups. If any recruitment materials are developed, they will not be distributed without IRB approval.

The subject population is derived from the greater Philadelphia area, which represents a racially and economically diverse population. We will make efforts for this protocol to be widely accessible, including offering the procedures protocol without charge to the subject.

## 3.3 Treatment Plan:

3.3.1 Informed consent will be obtained from all subjects before protocol specific activities are carried out. The subject will be informed about the limited data on oral supplementation and intravenous/oral NAC to support brain health in PD patients, possible risks and benefits, and possible adverse events. Informed consent will be documented by use of written consent form approved by the Institutional Review Board at Thomas Jefferson University and signed by the subject or the subject’s legal guardian. History, physical and neurological examination, and initial SPECT and MRS procedures (see below) will begin within 14 days of the informed consent process. Subjects will be receive either intravenous/oral NAC, or be placed on the waitlist control.

After approximately 90 days of receiving the supplements, subjects will undergo a follow up evaluation, which includes repeat neurological assessments, SPECT, and MRS. Note that subjects will continue taking the oral NAC until the scans are completed. Subjects will also continue to take their current Parkinson’s medication regimen so that they will continue to receive standard of care treatment throughout the study. It is hoped that their standard of care treatment will remain constant throughout the course of the study, but their referring neurologist may adjust their medications as medically necessary.

3.3.2 N-Acetylcysteine will be obtained from the Jefferson Pharmacy (NAC is also called Acetadote; Cumberland Pharmaceuticals). NAC is an intravenous (IV) medication for the treatment of acetaminophen overdose. Acetylcysteine is the nonproprietary name for the N-acetyl derivative of the naturally occurring amino acid, L-cysteine (N-acetyl-L-cysteine, NAC). Acetadote is supplied as a sterile solution in vials containing 200 mg/mL acetylcysteine. The pH of the solution ranges from 6.0 to 7.5. Acetadote contains the following inactive ingredients: 0.5 mg/mL disodium edetate, sodium hydroxide (used for pH adjustment), and Sterile Water for Injection, USP.

For the oral supplement, NAC is obtained by the Jefferson Pharmacy at 908 Walnut Street and supplied by Vital Nutrients (produced under GMP) as a 600mg capsule of N-acetyl cysteine. The following inactive ingredients are also included: Ascorbyl Palmitate, Gelatin, Rice, Silica. The capsules contain no coatings, binders, fillers, or dairy, wheat, eggs, soy, yeast, commercial sugars, starch, preservatives, or hydrogenated oil.

NAC doses will be prepared for each patient by the study nurse. The dose will be 50mg/kg in approximately 200ml of D5W infused over approximately one hour 1x per week. The IV bag containing NAC is labeled as a research medication. Subjects will also receive 600mg NAC tablets supplied by the Jefferson Pharmacy and will take 1 tablet 2x per day on the days that they do not receive the IV NAC.

## 3.4 Criteria for Removal from / Cessation of Protocol

3.4.1 Measuring Endpoints: Endpoints will be measured after receiving oral/intravenous NAC, or being on the waitlist for 90 days. Any serious adverse events also will result in immediate discontinuation of the subject from the study.

3.4.2 Subject Withdrawal: The subject may withdraw from the study at any time for any reason.

3.4.3 Missing Appointments: Missing a total of 5 or more days of the supplements or 5 doses of the intravenous NAC as per the PI.

All reasons for discontinuation of procedure will be documented in study flow sheets.

## 3.5 Adverse Events

The OHRP defines an adverse event as “any untoward or unfavorable medical occurrence in a human subject, including any abnormal sign, symptom, or disease, temporally associated with the subject’s participation in the research.” Adverse events can additionally be classified as an unanticipated problem, meaning it was not expected to occur during the course of the research. If an unexpected adverse event were to occur, it then needs to be determined whether or not it is due to the research being conducted. If the event is a result of the research procedures, most likely the event is directly connected to the subject’s participation in the research. It is also vital to determine whether an adverse event is serious. The OHRP defines a serious adverse event as one that a) results in death, b) is life-threatening, c) results in patient hospitalization or prolongation of existing hospitalization, d) results in persistent or significant disability/incapacity, e) results in congenital anomaly, or f) jeopardizes the subject’s health to the point where they may need medical or surgical intervention. If the adverse event is unexpected, related to the research, and serious (where it is causing harm to subjects), then it is also classified as an unanticipated problem and must be reported to the Thomas Jefferson University Hospital IRB. All adverse events will be reported in accordance with Jefferson IRB Adverse Events Report. The IRB shall be notified in a written safety report if any serious and unexpected adverse experience associated with the use of the oral or intravenous nutritional supplements occurs.

## 3.6 Data Collection and Submission Schedule

3.6.1 Data Submission: Data must be submitted according to the protocol requirements for all subjects registered, whether or not assigned treatment is administered.

3.6.2 Master files, such as case report forms and progress reports, are monitored by the study coordinator, Nancy Wintering. Case report forms will include eligibility checklist, demographic data, baseline history, adverse events, and off-study documents. These will be completed by study staff under the supervision of the investigators or the project manager.

### 3.7. Clinical Response

Subjects will be evaluated utilizing the UPDRS scores to determine any improvements in PD symptoms. Subjects will also be evaluated using the Profile of Moods Scale, Beck Depression Inventory, Mini-Mental Status Exam, and Parkinson’s disease Questionnaire-39. These will be obtained initially and after approximately 90 days of taking the supplements.

### 3.7.1 SPECT Imaging Procedure

(a) Subject Preparation - An indwelling catheter needle will be inserted into an antecubital vein. DaTSCAN will be administered through the indwelling line.

(b) DaTSCAN SPECT Imaging Procedure – Subjects will receive a standard of care DaTScan initially and after completing the NAC regimen. Subjects will be asked to arrive at the Nuclear Medicine SPECT Center on the day of the study. A signed informed consent form will be documented after all questions have been answered. Women of childbearing potential must have had a negative pregnancy test within 48 hours before proceeding with the SPECT study. The intravenous catheter will be inserted and capped. Approximately 30 minutes prior to injection of the DaTSCAN, the patient will be given an oral dose of lugol’s solution, which is the standard of care for these scans. Lugol’s solution helps to protect the thyroid from additional radioactive exposure from the DaTSCAN tracer. DaTSCAN (4-5 mCi, ± 20%) will be injected intravenously. After injection of the DaTSCAN, the venous catheter will be removed, and then the subject permitted to get off the examination table. Subjects can then take a break for lunch. Subjects will receive an MRI to assist with anatomic delineation. In addition, the MRI session will be utilized in order to obtain data via MR Spectroscopy (MRS), which can provide a measure of various metabolites, such as glutathione, in specific brain structures (please see below).

(c) Image Acquisition and Processing - The dose of DaTSCAN will be injected in an antecubital vein, and SPECT images acquired at approximately 3 hours post injection for about one hour. This will enable us to obtain quantitative regional uptake values as determined by our previously described reference region method. All scans will be performed on a Philips Forte gamma camera or equivalent camera equipped with ultra-high resolution collimators. All the images will be reconstructed using filtered back projection. Chang's first order correction method is used to compensate for photon attenuation.

Manual demarcation of brain regions - A set of standardized templates containing small ROIs will be fit on each DaTSCAN. Within the x-y plane, small ROIs in the template will be smaller than the actual structures they represent in order to minimize resolution-induced problems with ill-defined edges. To reduce the effects of volume averaging in the axial direction, the small ROIs will not be placed on the slices that contained the upper most and lower most portions of the structures they represent. This will tend to limit the small ROIs to the central aspect of structures they represent. The primary outcome measure will be the specific uptake values at 3 to 4 hours post administration, when the distribution of DaTSCAN has approached a transient, near equilibrium like state that reflects the ratio of k3/k4, which is related to binding potential. This allows for a quantitative assessment of dopamine transporter activity. Again, scanning at this time of a pseudo-equilibrium state allows for quantitative analysis using a region of interest analysis with a background region as an index of nonspecific binding.

As described above, it is probably not necessary to account for volumetric changes especially since the ROIs will be smaller than the actual structures. However, since all scans will have MRI correlation for adequate placement, volumes of the basal ganglia structures can be determined and utilized as an additional analysis. The reference region has typically been the cerebellum or the brain regions above the level of the basal ganglia, corresponding to areas of the brain, which have very low concentrations of DAT. However, values will be obtained for both whole brain regions above the basal ganglia and cerebellar regions in order to confirm the findings.

Statistical Parametric Mapping (SPM) will also be performed on the images in order to compare the pre- and post-intervention scans in PD subjects. SPM is currently considered to be a highly successful analytical tool for evaluating of SPECT brain images, and will be included in the analysis.

### 3.7.3 MR Spectroscopy Procedure

A brain proton magnetic resonance spectroscopic imaging (1H MRS) will be performed in the Department of Radiology at Thomas Jefferson University and Hospital over approximately one hour. The sections of the brain scanned will be oriented obliquely along the AC/PC line. Cortical and subcortical structures such as the basal ganglia will be evaluated for the following metabolites: glutathione; N-acetylaspartate (or NAA, an indicator of neuronal integrity), creatine+phosphocreatine (CR, important in cell energetics), choline (CHO, involved in cell membrane biosynthesis/degradation), and lactate (LAC, end product of glycolysis or anaerobic metabolism). Statistical Parametric Mapping will be used to determine the differences in the concentrations of the various metabolites before and after receiving intravenous/oral NAC. In addition, connectivity and resting cerebral blood flow can be measured during the same imaging session.

## 3.8 Statistical Considerations

3.8.1 Descriptive and Exploratory Analyses: The initial analyses of the data will be descriptive in nature. Using means, standard deviations, median, and range, the distribution volume ratios (DVRs) will be described for each time point before and after subjects have used the nutritional supplementation for approximately 90 days. Similarly, the clinical scores will be described for each time point. Graphical methods, such as plots of measurements over time, histograms, and boxplots are important tools for understanding the quality of the data, and assessing assumptions underlying statistical models (such as normality). Transformations will be applied as necessary to satisfy these assumptions. Plots of all of the measured variables over time will be important to assess longitudinal patterns of change.

3.8.2 Analyses for Primary Objectives: To evaluate whether intravenous/oral NAC helps to support dopamine function in the brain of patients with PD by measuring changes in the dopamine transporter. To accommodate the longitudinal nature of the data, a Heterogeneous Random Coefficients Model ([[62]](#endnote-63),[[63]](#endnote-64)) will be fit for the DVRs in each region for the DaTSCAN binding and each neurobehavioral score (i.e. UPDRS scores). This model can also account for both between and within subject heterogeneity and will accommodate modeling potentially nonlinear therapeutic effects over time. The random coefficients model is more flexible in terms of modeling the changes in DVRs and neuropsychological tests over time than repeated measures analyses of variance. The random coefficients model also has fewer restrictions on the correlation structure between multiple measures within subjects. ANCOVA will be used in order to determine if there are correlations between the pre-intervention neuropsychological and clinical measures and the longitudinal outcome measures after receiving oral or intravenous supplementation. This analysis will also evaluate whether the DaTSCAN DVRs can be utilized as a predictor for outcome. To determine if there is a correlation between changes in DAT binding and symptom severity, Pearson product-moment correlations will be computed unless the normality assumption is violated, in which case the Spearman rank correlation will be computed.

### 3.8.3 Image analysis with SPM:

Rationale for using SPM. The traditional method for analyzing brain SPECT images involves the placement of a number of ROI's on the image (as described above), and measuring the mean counts within that ROI. It has long been known that ROI definition, particularly on functional images, is time-consuming, subjective and prone to operator bias ([[64]](#endnote-65)). Small regular ROI's are capable of resolving localized changes between images, although they are subject to a loss of specificity due to problems associated with multiple comparisons. Alternatively, large ROI's encompassing an entire brain structure may dilute small activation foci with a subsequent loss of sensitivity. Unless a study is strongly hypothesis driven, and comparisons between images are *only* made in highly specific regions; ROI analysis lacks the power to distinguish regional variations between a set of images accurately. The solution to this problem has been the development of pixel-based statistical analyses where the regions are effectively reduced to single pixels ([[65]](#endnote-66)). Our group has applied SPM, and other voxel-based techniques, to cerebral blood flow and binding site studies previously ([[66]](#endnote-67)) and has considerable experience in these methods.

SPM analysis. A typical SPM analysis of a set of brain scans can be reduced to three essentially independent steps: image normalization, statistical analysis, and significance assessment. Once images are normalized to a standard template, pixel-by-pixel statistical tests are performed comparing the individual scans and correcting for inter-scan and inter-subject variability. This leads to the generation of (uncorrected) parametric images of significant differences. In the absence of absolute quantification, an important feature of SPM is the method of normalizing each individual scan to prevent inter-subject and inter-scan variability masking regional changes. The regional activity is normalized to a reference value derived independently from each image. This reference must accurately reflect an unbiased baseline of activity not affected by the disease or activation under study. For cerebral binding sites, the normalizing factor is usually a measure of background activity such as counts in the cerebellum. This is the background region used in the equilibrium model of DaTSCAN binding, giving a semi-quantitative measure of tracer uptake that is proportional to the binding potential.

Significance assessment. The statistical parametric maps represent significant differences at the pixel-level. However, in a typical brain image, there are many thousands of pixels, and the probability that many of them will be significant purely by chance is high, even for tight thresholds. The final step in SPM assesses the significance of *clusters* of suprathreshold pixels, correcting for the effects of such a large number of correlated multiple comparisons using the theory of random Gaussian fields. Statistically significant differences between and baseline and post-treatment scans will be assessed at each voxel with a threshold of P < 0.001. To correct for correlated multiple comparisons, clusters of voxels which survive this threshold will be assessed further using the theory of random Gaussian fields ([[67]](#endnote-68),[[68]](#endnote-69)), which calculates the significance of clusters based on their peak height and spatial extent. Due to the limited volume of the striatum, the small volume correction will be used within SPM, allowing a more sensitive test. With this technique, differences in DaTSCAN binding between baseline and post-treatment scans will be assessed, and statistic images generated showing significant regional differences. A correlational analysis with various neuropsychological parameters such as the UPDRS, profile of mood scale, and Parkinson’s Disease Questionnaire will also be performed with SPM. Similar analysis will be performed for the MRS data, which will compare the concentration of the specific molecules measured by MRS before and after three months of the nutritional supplementation program.

3.8.4 Power Analysis: We view this study primarily as a pilot study to assess changes in physiological and clinical measures and determine the effect size for powering future, larger trials. However, we estimated the sample size needed based upon prior research we have performed evaluating DAT imaging in the study of PD patients. We have previously found that the PD patients can have variability in their DVR measures of dopamine binding of approximately 10%. In reviewing the current data, we have found approximately a 7% difference between the NAC and control group. If we expect a 7% improvement in the NAC group, for an 80% power to detect a significant change of p=0.05, we would need approximately 33 subjects in the treatment arms and approximately 33 subjects in the control arm. The statistical analyses for the proposed study will consist of 1) repeated measures two-way ANOVA, and 2) linear regression. Both methods are more powerful than simple t-tests on which these estimates are based. Thus, we plan to recruit 33 subjects in the intravenous/oral NAC group, and 33 subjects in the waitlist control group. People randomized to the control group will be given the option to re-enroll in the study and receive the NAC.

### 3.8.5 Randomization: Randomization will occur via a 1:1 ratio of the,intravenous/oral NAC, or waitlist control groups using the method of random permuted blocks with random block sizes without stratification.

# 4.0 RISKS

**4.1 N-acetyl cysteine:** Oral NAC has few side-effects and is commonly used as an over-the-counter supplement worldwide. Oral NAC has good, but variable absorption from the gut ([[69]](#endnote-70),[[70]](#endnote-71)), making it problematic as the sole mode of supplementation in a clinical study. Injectable NAC is used at lower doses for exercise fatigue and higher doses primarily as a liver protector. The dosages in this study are consistent with those used in the exercise physiology literature ([[71]](#endnote-72)). Side effects increase with higher dosage, and the most common associated adverse reactions in the literature attributed to IV NAC administration are rash, urticaria, and pruritus. The frequency of adverse events has been reported to be between 0.2% and 20.8%, and they most commonly occur during the initial loading dose of acetylcysteine at dosages higher than what will be used in this study. Other side effects with greater than 1% occurrence include nausea and bronchospasm, again at higher dosages. A hypersensitivity reaction to NAC has been reported as a rare occurrence. NAC should be used with caution in patients with asthma. Please note that the oral and intravenous NAC dose used in this study is the same as one we have used in a study of NAC in women with breast cancer. We have found the NAC to be generally well tolerated at the doses we will be using in the current study.

## **4.2 Oral NAC/intravenous Supplements:**

### **4.2.1 Supplements, Doses, and Risks**

General Risks: No formal interaction studies have been performed in the literature. Thus, it is possible that they may interact with each other and produce side effects as described below or additional side effects. Any supplement could result in an allergic reaction. We will ask subjects to report any unusual feelings they have while receiving the supplements. In addition, the supplements could interact with different medications. We will evaluate to determine if patients are taking any of the following:

1. Medications for high blood pressure.

2. Medications that slow blood clotting.

3. Medications for diabetes.

4. Nitroglycerin.

We will review each patient’s medications to determine if there may be some potential risk for taking the NAC. For medications used for moderate to severe conditions as determined by the PI, subjects will be excluded. For medications used for mild conditions, we will discuss with the patient and also their doctor how to closely monitor any changes in their response to the medications while on the study.

**4.3 Potential Risks of DaTScan:** The I-123 DaTSCAN is a commercially available radioactive tracer that will be used according to its dose, route, and indication, but results in some exposure to ionizing radiation. The amount is acceptable for the research subjects who will directly benefit by receiving full clinical reads of these scans that their referring physician can utilize for determination of prognosis and treatment planning. Subjects will be required to lie still on the imaging table for 30-60 minutes, which can be uncomfortable.

**4.4 Special Risk Factors:** Injection of DaTscan requires the routine clinical pre-administration of Lugol’s solution in order to protect the thyroid from radioactive exposure. The Lugol’s solution is usually given in juice; it sometimes causes people to have a temporary strange taste in their mouth or a feeling of discomfort in their salivary glands.

**4.5 Risks of venous cannulation:** Venous cannulation is a routine clinical procedure that carries minimal risks when performed by trained personnel. It is possible that bruising could occur in some subjects. There is a theoretical risk of phlebitis or infection, which is very remote.

**4.6 Magnetic Resonance Spectroscopy:** MRS is performed in an MRI scanner, requires a magnetic field. MRS can be dangerous if a person has metal or metallic objects in their body. Subjects will be thoroughly screened to ensure that they have no metal in their body. Because of the magnetic field, metallic objects can move into the scanner and potentially injure the patient. All precautions are taken to ensure that no such metallic objects are in the scanning room that could result in an injury. The MRS requires the patient to lie still for approximately 1 hour, which can be uncomfortable, or be claustrophobic.

# 5.0 REFERENCES

1. . Sies H, Cadenas E. Oxidative stress: damage to intact cells and organs. Philos Trans R Soc Lond B Biol Sci 1985;311:617–631. [↑](#endnote-ref-2)
2. . Chinta SJ, Andersen JK. Redox imbalance in Parkinson's disease. Biochimica et Biophysica Acta 2008;1780: 1362–1367 [↑](#endnote-ref-3)
3. . Jenner P. Oxidative stress in Parkinson's disease. Ann Neurol 2003;53:S26–S36 discussion S36–28. [↑](#endnote-ref-4)
4. . Jenner P, Olanow CW. Oxidative stress and the pathogenesis of Parkinson's disease. Neurology 1996;47:S161–S170. [↑](#endnote-ref-5)
5. . Youdim MB, Riederer P. Understanding Parkinson's disease. Sci Am 1997;276:52–59. [↑](#endnote-ref-6)
6. . Scherman D, Desnos C, Darchen F, Pollak P, Javoy-Agid F, Agid Y. Striatal dopamine deficiency in Parkinson's disease: role of aging, Ann Neurol 1989; 26: 551–557. [↑](#endnote-ref-7)
7. . Jenner P Presymptomatic detection of Parkinson's disease. J Neural Transm 1993;Suppl. 40: 23–36. [↑](#endnote-ref-8)
8. . Jenner P, Olanow CW. Oxidative stress and the pathogenesis of Parkinson's disease. Neurology 1996;47:S161–S170. [↑](#endnote-ref-9)
9. . Kaur D, Andersen J. Does cellular iron dysregulation play a causative role in Parkinson's disease? Ageing Res Rev 2004;3:327–343. [↑](#endnote-ref-10)
10. . Lan J, Jiang DH. Desferrioxamine and vitamin E protect against iron and MPTP induced neurodegeneration in mice J Neural Trans 1997;104:469–481. [↑](#endnote-ref-11)
11. . Grunblatt E, Mandel S, Berkuzki T, Youdim MB. Apomorphine protects against MPTP-induced neurotoxicity in mice. Mov Disord 1999; 14; 612–618. [↑](#endnote-ref-12)
12. . Kaur D, Yantiri F, Rajagopalan S, et al. Genetic or pharmacological iron chelation prevents MPTP-induced neurotoxicity in vivo: a novel therapy for Parkinson's disease. Neuron. 2003 Mar 27;37(6):899-909. [↑](#endnote-ref-13)
13. . Braak H, Braak E. Pathoanatomy of Parkinson's disease. J Neurol 2000;247 (Suppl 2): II3–10. [↑](#endnote-ref-14)
14. . Paxinou E, Chen Q, Weisse M, Giasson BI, Norris EH Rueter SM, Trojanowski J, Lee VM, Ischiropoulos H. Induction of alpha-synuclein aggregation by intracellular nitrative insult. J Neurosci 2001; 21:8053–8061. [↑](#endnote-ref-15)
15. . Ischiropoulos H, Beckman JS. Oxidative stress and nitration in neurodegeneration: cause, effect, or association? J Clin Invest 2003;11:163–169. [↑](#endnote-ref-16)
16. . Uversky VN, Li J, Fink AL. Metal-triggered structural transformations, aggregation, and ﬁbrillation of human α-synuclein. J Biol Chem 2001;23:44284–44296. [↑](#endnote-ref-17)
17. . Hashimoto M, Hsu LJ, Xia Y, Takeda A, Sisk A, Sundsmo M, Masliah E. Oxidative stress induces amyloid-like aggregate formation of NACP/alpha-synuclein in vitro, Neuroreport 1999;10:717–721. [↑](#endnote-ref-18)
18. . Thomas MP, Chartrand K, Reynolds A, Vitvitsky V, Banerjee R, Gendelman HE. Ion channel blockade attenuates aggregated alpha synuclein induction of microglial reactive oxygen species: relevance for the pathogenesis of Parkinson's disease. J Neurochem 2007; 100:503–519. [↑](#endnote-ref-19)
19. . Cobley JN, McGlory C, Morton JP, Close GL. N-Acetylcysteine's attenuation of fatigue after repeated bouts of intermittent exercise: practical implications for tournament situations. Int J Sport Nutr Exerc Metab. 2011 Dec;21(6):451-61. [↑](#endnote-ref-20)
20. . Medved I, Brown MJ, Bjorksten AR, Murphy KT, Petersen AC, Sostaric S, Gong X, McKenna MJ. N-acetylcysteine enhances muscle cysteine and glutathione availability and attenuates fatigue during prolonged exercise in endurance-trained individuals. J Appl Physiol. 2004 Oct;97(4):1477-85. [↑](#endnote-ref-21)
21. . Jiang Y, Rumble JL, Gleixner AM, et al. N-Acetyl cysteine blunts proteotoxicity in a heat shock protein-dependent manner. Neuroscience. 2013 Dec 26;255:19-32. [↑](#endnote-ref-22)
22. . Unnithan AS, Jiang Y, Rumble JL, et al. N-Acetyl cysteine prevents synergistic, severe toxicity from two hits of oxidative stress. Neurosci Lett. 2014 Feb 7;560:71-6. [↑](#endnote-ref-23)
23. . R.R. Ratan, T.H. Murphy, J.M. Baraban. Macromolecular synthesis inhibitors prevent oxidative stress-induced apoptosis in embryonic cortical neurons by shunting cysteine from protein synthesis to glutathione. J Neurosci, 17 (1994), pp. 4385–4392. [↑](#endnote-ref-24)
24. . J. Clark, E.L. Clore, K. Zheng et al. Oral N-acetyl-cysteine attenuates loss of dopaminergic terminals in α-synuclein over-expressing mice. PLoS One, 5 (2010), p. e12333. [↑](#endnote-ref-25)
25. . E. Aoki, R. Yano, H. Yokoyama, H. Kato, T. Araki. Role of nuclear transcription factor kappa B (NF-kappaB) for MPTP (1-methyl-4-phenyl-1,2,3,6-tetrahyropyridine)-induced apoptosis in nigral neurons of mice. Exp Mol Pathol, 86 (2009), pp. 57–64 [↑](#endnote-ref-26)
26. . D. Sha, L.S. Chin, L. Li. Phosphorylation of parkin by Parkinson disease linked kinase PINK1 activates parkin E3 ligase function and NF-kappaB signaling. Hum Mol Genet, 19 (2010), pp. 352–363. [↑](#endnote-ref-27)
27. . M.B. Bagh, A.K. Maiti, S. Jana. et al. Quinone and oxyradical scavenging properties of N-acetylcysteine prevent dopamine mediated inhibition of Na+, K+-ATPase and mitochondrial electron transport chain activity in rat brain: implications in the neuroprotective therapy of Parkinson’s disease. Free Radic Res, 42 (2008), pp. 574–581 [↑](#endnote-ref-28)
28. . Chandramani Shivalingappa P, Jin H, Anantharam V, Kanthasamy A, Kanthasamy A. N-Acetyl Cysteine Protects against Methamphetamine-Induced Dopaminergic Neurodegeneration via Modulation of Redox Status and Autophagy in Dopaminergic Cells. Parkinsons Dis. 2012;2012:424285. [↑](#endnote-ref-29)
29. . M. Martínez-Banaclocha, A.I. Hernández, N. Martínez et al. N-acetylcysteine protects against age-related increase in oxidized proteins in mouse synaptic mitochondria. Brain Res, 762 (1997), pp. 256–258. [↑](#endnote-ref-30)
30. . J. Vina, F.J. Romero, G.T. Saez, et al. Effects of cysteine and N-acetyl cysteine on GSH content of brain of adult rats. Experientia, 39 (1983), pp. 164–165. [↑](#endnote-ref-31)
31. . Martínez-Banaclocha M, Martínez N, Hernández, et al. Hypothesis: can N-acetylcysteine be beneficial in Parkinson’s disease? Life Sci 1999;64:1253–7. [↑](#endnote-ref-32)
32. . M. Martínez-Banaclocha. N-acetylcysteine elicited increase in complex I activity in synaptic mitochondria from aged mice. Implications for treatment of Parkinson’s disease. Brain Res, 859 (2000), pp. 173–175 [↑](#endnote-ref-33)
33. . T.L. Perry, V.W. Yong, R.M. Clavier et al. Partial protection from the dopaminergic neurotoxin Nmethyl-4-phenyl-1,2,3,6-tetrahydropyridine by four different antioxidants in the mouse. Neurosci Lett, 60 (1985), pp. 109–114. [↑](#endnote-ref-34)
34. . A. Sharma, P. Kaur, V. Kumar et al. Attenuation of 1-methyl-4-phenyl-1, 2,3,6-tetrahydropyridine induced nigro-striatal toxicity in mice by N-acetyl cysteine. Cell Mol Biol, 53 (2007), pp. 48–55. [↑](#endnote-ref-35)
35. . S. Medina, M. Martínez-Banaclocha, A. Hernanz. Antioxidants inhibit the human cortical neuron apoptosis induced by hydrogen peroxide, tumor necrosis factor alpha, dopamine and beta-amyloid peptide 1–42. Free Radic Res, 36 (2002), pp. 1179–1184. [↑](#endnote-ref-36)
36. . J. Clark, E.L. Clore, K. Zheng, et al. Oral N-acetyl-cysteine attenuates loss of dopaminergic terminals in α-synuclein over-expressing mice. PLoS One, 5 (2010), p. e12333 [↑](#endnote-ref-37)
37. . Berman AE, Chan WY, Brennan AM, et al. N-acetylcysteine prevents loss of dopaminergic neurons in the EAAC1-/- mouse. Ann Neurol. 2011 Mar;69(3):509-20. [↑](#endnote-ref-38)
38. . Holmay MJ, Terpstra M, Coles LD, et al. N-acetylcysteine boosts brain and blood glutathione in Gaucher and Parkinson diseases. Clin Neuropharmacol. 2013 Jul-Aug;36(4):103-6. [↑](#endnote-ref-39)
39. . Hauser RA, Lyons KE, McClain T, Carter S, Perlmutter D. Randomized, double-blind, pilot evaluation of intravenous glutathione in Parkinson's disease. Mov Disord 2009;24(7):979-83. [↑](#endnote-ref-40)
40. . Giros B, Caron MG: Molecular characterization of the dopamine transporter. Trends Pharm Sci 1993; 14:43-49. [↑](#endnote-ref-41)
41. . Jaber M. Jones S. Giros B. Caron MG. The dopamine transporter: a crucial component regulating dopamine transmission. Movement Disorders 1997; 12:629-633. [↑](#endnote-ref-42)
42. . Giros B, Jaber M, Jones SR, Wightman RM, Caron MG. Hyperlocomotion and indifference to cocaine and methamphetamine in mice lacking the dopamine transporter. Nature 1996; 379:606-612. [↑](#endnote-ref-43)
43. . Frost JJ, Rosier AJ, Reich SG, Smith JS, Ehlers MD, Snyder SH, Ravert HT, Dannals RF. Positron emission tomographic imaging of the dopamine transporter with 11C-WIN 35,428 reveals marked decline in mild Parkinson's disease. Ann Neurol 1993; 34:423-431. [↑](#endnote-ref-44)
44. . Shoemaker H, Pimoule C, Arbilla S, Scatton B, Javoy-Agid F, Langer SZ. Sodium dependent [3H] cocaine binding associated with dopamine uptake sites in the rat striatum and human putamen decrease after dopaminergic denervation and in Parkinson's disease. Naunyn-Schmiedeberg's Arch Pharmacol 1985; 239:227-235. [↑](#endnote-ref-45)
45. . Innis RB, Seibyl JP, Scanley BE, et al. Single-photon emission-computed tomography imaging demonstrates loss of striatal dopamine transporters in Parkinson disease. Proc Natl Acad Sci USA 1994. [↑](#endnote-ref-46)
46. . Donnan GA, Woodhouse DG, Kaczmarczyk SJ, et al. Evidence for plasticity of the dopaminergic system in Parkinsonism. Molec Neurobiol 1991; 5:421-433. [↑](#endnote-ref-47)
47. . Seibyl JP, Marek K, Sheff K, et al. Iodine-123-beta-CIT and iodine-123-FPCIT SPECT measurement of dopamine transporters in healthy subjects and Parkinson's patients. J Nucl Med 1998;39(9):1500-8. [↑](#endnote-ref-48)
48. . Eshuis SA, Jager PL, Maguire RP, et al. Direct comparison of FP-CIT SPECT and F-DOPA PET in patients with Parkinson's disease and healthy controls. Eur J Nucl Med Mol Imaging 2009;36(3):454-62. [↑](#endnote-ref-49)
49. . Ceravolo R, Antonini A, Volterrani D, et al. Predictive value of nigrostriatal dysfunction in isolated tremor: a clinical and SPECT study. Mov Disord 2008;23(14):2049-54. [↑](#endnote-ref-50)
50. . Varrone A, Sansone V, Pellecchia MT, et al. Comparison between a dual-head and a brain-dedicated SPECT system in the measurement of the loss of dopamine transporters with [123I]FP-CIT. Eur J Nucl Med Mol Imaging 2008;35(7):1343-9. [↑](#endnote-ref-51)
51. . Booij J, Tissingh G, Boer GJ, et al. [123I]FP-CIT SPECT shows a pronounced decline of striatal dopamine transporter labelling in early and advanced Parkinson's disease. J Neurol Neurosurg Psychiatry 1997;62(2):133-40. [↑](#endnote-ref-52)
52. . Wang J, Zuo CT, Jiang YP, et al. 18F-FP-CIT PET imaging and SPM analysis of dopamine transporters in Parkinson's disease in various Hoehn & Yahr stages. J Neurol 2007;254(2):185-90. [↑](#endnote-ref-53)
53. . Hesse S, Strecker K, Winkler D, et al. Effects of subthalamic nucleus stimulation on striatal dopaminergic transmission in patients with Parkinson's disease within one-year follow-up. J Neurol 2008;255(7):1059-66. [↑](#endnote-ref-54)
54. . Parkinson Study Group. Dopamine transporter brain imaging to assess the effects of pramipexole vs. levodopa on Parkinson disease progression. JAMA 2002;287(13):1653-61. [↑](#endnote-ref-55)
55. . Clarke CE, Lowry M. Systematic review of proton magnetic resonance spectroscopy of the striatum in parkinsonian syndromes. Eur J Neurol 2001;8:573-577. [↑](#endnote-ref-56)
56. . Cruz CJ, Aminoff MJ, et al. Proton MR spectroscopic imaging of the striatum in Parkinson’s disease. Magn Reson Imaging 1997; 15(6):619-624 [↑](#endnote-ref-57)
57. . Lucetti C, Del Dotto P, Gambaccini G, Bernardini S, Bianchi MC, Tosetti M, Bonuccelli U. Proton magnetic resonance spectroscopy (1H-MRS) of motor cortex and basal ganglia in de novo Parkinson's disease patients. Neurol Sci 2001;22(1):69-70. [↑](#endnote-ref-58)
58. . Lucetti C, Del Dotto P, Gambaccini G, et al. Influences of dopaminergic treatment on motor cortex in Parkinson disease: a MRI/MRS study. Mov Disord 2007;22(15):2170-5. [↑](#endnote-ref-59)
59. . Harris JL, Yeh HW, Choi IY, et al. Altered neurochemical profile after traumatic brain injury: (1)H-MRS biomarkers of pathological mechanisms. J Cereb Blood Flow Metab 2012. doi: 10.1038/jcbfm.2012.114. [Epub ahead of print] [↑](#endnote-ref-60)
60. . An L, Dani KA, Shen J, Warach S. Pilot results of in vivo brain glutathione measurements in stroke patients. J Cereb Blood Flow Metab 2012. doi: 10.1038/jcbfm.2012.127 [↑](#endnote-ref-61)
61. . Mandal PK, Tripathi M, Sugunan S. Brain oxidative stress: detection and mapping of anti-oxidant marker 'Glutathione' in different brain regions of healthy male/female, MCI and Alzheimer patients using non-invasive magnetic resonance spectroscopy. Biochem Biophys Res Commun 2012;417(1):43-8. [↑](#endnote-ref-62)
62. . Byrk AS and Raudenbush SW. Hierarchical linear models. Newbury Park, CA: Sage Publications, Inc., 1992. [↑](#endnote-ref-63)
63. . Litell RC, Millikin GA, Stroup WW, Wolfinger RD. SAS system for mixed models. Carey, NC: SAS Institute; 1996. [↑](#endnote-ref-64)
64. . Acton PD, Friston KJ. Statistical parametric mapping in functional neuroimaging: beyond PET and fMRI activation studies. Eur J Nucl Med 1998; 25:663-667. [↑](#endnote-ref-65)
65. . Friston KJ, Holmes AP, Worsley KJ, Poline JP, Frith CD, Frackowiak RSJ. Statistical parametric maps in functional imaging: a general linear approach. Hum Brain Map 1995; 2:189-210. [↑](#endnote-ref-66)
66. . Acton PD, Mozley PD, Kung HF. Logistic discriminant parametric mapping: a novel method for the pixel-based differential diagnosis of Parkinson's disease. Eur J Nucl Med 1999; 26: 1413-1423. [↑](#endnote-ref-67)
67. . Worsley KJ, Marrett S, Neelin P, Vandal AC, Friston KJ, Evans AC. A unified statistical approach for determining significant signals in images of cerebral activation. Hum Brain Map 1996; 4:58-73. [↑](#endnote-ref-68)
68. . Friston KJ, Worsley KJ, Frackowiak RSJ, Mazziotta JC, Evans AC. Assessing the significance of focal activations using their spatial extent. Hum Brain Map 1994; 1:214-220. [↑](#endnote-ref-69)
69. . Borgstrom L, Kagedal B, Paulsen O. Pharmacokinetics of N-acetylcysteine in man. Eur J Clin Pharmacol 1986;31:217-222. [↑](#endnote-ref-70)
70. . De Caro L, Ghizzi A, Costa R, et al. Pharmacokinetics and bioavailability of oral acetylcysteine in healthy volunteers. Arzneim Forsch 1989;39:382-385. [↑](#endnote-ref-71)
71. . Sen CK, Packer L. Thiol homeostasis and supplements in physical exercise. Am J Clin Nutr. 2000 Aug;72(2 Suppl):653S-69S. [↑](#endnote-ref-72)
